# Examining the effectiveness of place-based interventions to improve public health and reduce health inequalities: an umbrella review

**DOI:** 10.1186/s12889-021-11852-z

**Published:** 2021-10-19

**Authors:** V J McGowan, S. Buckner, R. Mead, E. McGill, S. Ronzi, F. Beyer, C. Bambra

**Affiliations:** 1 Population Health Sciences Institute, Newcastle University, 5th Floor, Ridley 1, Newcastle Upon Tyne, NE1 7RU UK; 2 Fuse – The Centre for Translational Research in Public Health, Newcastle Upon Tyne, UK; 3 Cambridge Public Health, University of Cambridge, Cambridge, UK; 4 Department of Health Research, Lancaster University, Lancaster, UK; 5 LiLaC – Liverpool and Lancaster Universities Collaboration for Public Health Research, Lancaster, UK; 6 Department of Health Services Research and Policy, London School of Hygiene and Tropical Medicine, London, UK; 7 Department of Public Health and Policy, University of Liverpool, Liverpool, UK

**Keywords:** Place, Health, Inequalities, Economic uncertainty

## Abstract

**Background:**

Locally delivered, place-based public health interventions are receiving increasing attention as a way of improving health and reducing inequalities. However, there is limited evidence on their effectiveness. This umbrella review synthesises systematic review evidence of the health and health inequalities impacts of locally delivered place-based interventions across three elements of place and health: the physical, social, and economic environments.

**Methods:**

Systematic review methodology was used to identify recent published systematic reviews of the effectiveness of place-based interventions on health and health inequalities (PROGRESS+) in high-income countries. Nine databases were searched from 1st January 2008 to 1st March 2020. The quality of the included articles was determined using the Revised Assessment of Multiple Systematic Reviews tool (R-AMSTAR).

**Results:**

Thirteen systematic reviews were identified - reporting 51 unique primary studies. Fifty of these studies reported on interventions that changed the physical environment and one reported on changes to the economic environment. Only one primary study reported cost-effectiveness data. No reviews were identified that assessed the impact of social interventions. Given heterogeneity and quality issues, we found tentative evidence that the provision of housing/home modifications, improving the public realm, parks and playgrounds, supermarkets, transport, cycle lanes, walking routes, and outdoor gyms – can all have positive impacts on health outcomes – particularly physical activity. However, as no studies reported an assessment of variation in PROGRESS+ factors, the effect of these interventions on health inequalities remains unclear.

**Conclusions:**

Place-based interventions can be effective at improving physical health, health behaviours and social determinants of health outcomes. High agentic interventions indicate greater improvements for those living in greater proximity to the intervention, which may suggest that in order for interventions to reduce inequalities, they should be implemented at a scale commensurate with the level of disadvantage. Future research needs to ensure equity data is collected, as this is severely lacking and impeding progress on identifying interventions that are effective in reducing health inequalities.

**Trial registration:**

PROSPERO CRD42019158309

**Supplementary Information:**

The online version contains supplementary material available at 10.1186/s12889-021-11852-z.

## Background

The links between places, communities and health have long been established in the scientific literature [1, 2]. Differences in ‘place’ characteristics can also help to explain why there are large and persistent inequalities in health between places. In England, for example, healthy life expectancy varies by 21.5 years for women and 15.8 years for men between local government areas while even wider disparities exist between smaller areas [3]. Similarly, these localised inequalities in health are experienced in other high-income countries. The capital of the United States (US), Washington DC, has a 20 year gap in life expectancy between low income and more affluent neighbourhoods [2]. Place-based health inequalities are also noted across Europe, with regional north/south health inequalities persisting in areas such as Italy [4] and Spain [5], and some of the highest regional levels of inequalities found in France, Germany, the United Kingdom (UK), and Austria [6].

These geographical inequalities in health are often attributed to: compositional and contextual aspects of place and their interelationship [7, 8]. Compositional explanations ascribe differences in health between places to the characteristics of individuals who live in these areas [9], whereas contextual explanations attribute differences in health to differences in the characteristics of places where people live [7]. The relational approach to health and place notes their interactions between these two levels – as the characteristics of individuals (compositional) are influenced by the characteristics of the area (contextual) [10]. For example, the health effects of individual deprivation, such as lower income, can be amplified by area deprivation: ‘amplification of deprivation’. [11] There are numerous reviews of the literature examining the effects of compositional based interventions (e.g. alcohol and substance misuse, weight loss, diet and physical activity [12–15]) on the health of individuals and, indeed, many public health interventions are focused on changing the behaviour of individuals, for example, weight management, smoking cessation, and alcohol and drug services [16, 17]. However, there is evidence to suggest that compositional interventions require high levels of individual agency to accrue health benefits and differences in personal resources may lead to these interventions reinforcing or exacerbating inequalities in health [18, 19]. More recently, systematic reviews have assessed interventions that sought to modify the impact of contextual factors on health that have focused on large-scale, nationally funded, urban regeneration, housing-led, or child-focused interventions [20–24]. However, these programmes are not always delivered in areas of greatest need, potentially widening regional inequalities in health [25–27]. A more recent framing of the relationship between health and place is the political economy one [28]. It ‘scales-up’ the contextual explanation by highlighting the importance of macro-economic, political choices and public policies (such as austerity) in shaping both place *and* health - relationally [8]. Reviews examining these types of interventions suggest that market regulation of tobacco, alcohol and food is likely to be effective at improving health and reducing inequalities in health [29].

Local governments also seek to improve population health and reduce health inequalities through different kinds of improvements to the places people live in and frequent [20–23]. For example, Macintyre et al. (2009) [24] describe five broad types of socio-environmental influences on health covering many elements that fall within the remit of local governments; 1) physical features of the environment such as air and water quality, climate and latitude; 2) availability of healthy/unhealthy environments at home, work, and play including decent housing, secure and non-hazardous employment, affordable and nutritious food, and safe and healthy recreational facilities and activities; 3) access to services such as transport, education, policing, street cleaning and lighting, religious and community organisations, and health and welfare services; 4) socio-cultural features, political, economic, ethnic and religious history, community norms and values, level of community cohesion, perception of crime and safety, and community support networks; and 5) internal and external perceptions of place and place-related stigma [24, 30]. These factors highlight how local government has a potential wide-ranging role to play in delivering ‘place-based’ approaches because many of its functions directly and indirectly affect social determinants of health and wellbeing: planning, transport, high street regulation, housing, education, social work, children, and, in many countries (including England), public health itself.

Although local governments have some powers to shape local environments and promote health (varying by country) – and are increasingly being encouraged to do so, a number of factors limit the delivery of community and place-based interventions. These include the limited statutory powers over the social determinants of health (e.g. planning decisions or schools) but most notably, local governments in England and some other parts of Europe (e.g. Greece, Spain and Portugal) have been operating under increased financial constraints since the implementation of austerity measures as a result of the global financial crisis in 2008 [31, 32].

Research on place-based public health interventions, therefore, is timely, especially in light of the COVID-19 pandemic (given the high geographical inequalities in COVID-19 – associated with inequalities in chronic diseases [33]), international economic uncertainty, and acknowledgement amongst policymakers of the need to intensify our focus on primary prevention post-pandemic [33, 34]. The latter requires an understanding of the current evidence base. Therefore, there is a need to examine the effectiveness of locally-delivered place-based approaches to improving/maintaining (a) the physical environment (e.g. active travel, pedestrianisation, school crossing patrols, green space, cycle/walking routes, playgrounds, outdoor gyms, air pollution, fly-tipping/littering, housing); (b) the social environment (e.g. children’s services, alcohol and food licensing powers, provision of health promotion services, cultural venues/activities); and (c) the economic environment (e.g. local investment and growth strategies including local employment/training/education, subsidised public transport, welfare such as council tax discounts, and economic development initiatives).

The challenge of how to deliver effective place-based interventions to improve health and reduce inequalities in the current circumstances is not simply an economics question. There are already studies that show public health prevention saves costs on future health care and can also reduce other potential costs (e.g. crime, workplace absenteeism, productivity) [35–37]. However, we know relatively little about the different options available for delivering place-based strategies to improve health and reduce health inequalities when resources are limited and uncertain. Therefore, this umbrella review locates, appraises, and synthesises evidence of effectiveness from existing systematic reviews and considers locally delivered place-based interventions across three key elements of place and health; the physical, social, and economic environment [10].

### Research question

What place-based interventions are effective in improving health and reducing health inequalities?

## Methods

### Registration

This study is registered in PROSPERO as CRD42019158309 [38].

### Study design

Systematic review methodology was used to locate, appraise, and synthesise published systematic review-level evidence on the effectiveness of place-based interventions on health and health inequalities. The review followed established guidelines for the conduct and reporting of systematic reviews [39–41] and adhered to the Preferred Reporting Items for Systematic Reviews and Meta-Analysis (PRISMA) statement guidelines [42] (see Additional file 1 for completed PRISMA checklist). This umbrella review provides a high-level overview of existing reviews and is part of a wider study that aimed to review all available published evidence regardless of study design. Therefore, the results will be presented in three interlinked papers reporting the qualitative, and quantitative evidence in primary studies, and systematic review evidence. In this paper we report on the results of the umbrella review section of the study. Umbrella reviews − overviews of systematic reviews − build on the strengths of individual reviews and add scale by integrating the findings of multiple reviews together [43]. It is an established and well used method within public health research [29, 44–46]. The full methodology has been previously described in the published protocol [38].

### Search strategy

A search strategy was designed by an experienced information specialist (FB) in consultation with members of the research team, public and practice-based stakeholders, and with reference to previous umbrella search strategies (e.g. Thomson et al., 2018) [47].

The search strategy was designed in MEDLINE (see Additional file 1). The strategy used thesaurus headings and terms in the title, abstract and keyword fields, and was translated to other databases as appropriate. For the place-based element of the search, synonyms were used for place-based, for urban renewal or regeneration, and for elements of the physical environment including home, work, and recreational. The concept of health or health inequalities and associated synonyms comprised the second element, and finally a validated filter was used to exclude studies based in low and middle income countries [48]. We limited studies to high income countries for several reasons. Firstly, policy and practice colleagues indicated during project development that they required evidence of effectiveness for place-based interventions that would be comparable to their settings. Given low-middle income countries often lack universal health care and have limited planning and infrastructure [49] available to implement such interventions we limited our searches to high income countries in order to retrieve relevant and generalisable evidence. Although we recognise generalisability is still problematic across high income countries [50].

Changes were made to the strategy cited in the protocol to give a better balance of sensitivity and specificity. The search was run on the following nine databases for studies published between 1st Jan 2008 and 1st March 2020: Medline (Ovid); Scopus (Elsevier); Embase (Ovid); International Bibliography of the Social Sciences (IBSS; ProQuest); Sociological Abstracts (ProQuest); Social Services Abstracts (ProQuest) (included with Sociological Abstracts); Cochrane Database of Systematic Reviews and CENTRAL (Cochrane Library, Wiley); Social Care Online (SCIE); and Cumulative Index of Nursing and Allied Health Literature (CINAHL; EBSCOhost). The search was restricted to English language studies. The search start date of 2008 was driven by several factors. Our review was co-produced with public health policy and practice colleagues who wanted to understand what interventions were effective at improving health and reducing inequalities in austere times as local authority budgets had been decreasing since the global financial crisis [31, 51–55]. Additionally, previous evidence in this area (e.g. Bambra et al., 2010; Gibson et al., 2011; Larsen, 2007) [23, 56, 57] report pre-2008 studies and required updating to reflect changes in the economic environment.

In addition, we conducted citation follow-up and a grey literature search via Open Grey (http://www.opengrey.eu/) to identify any additional studies. We also conducted a consultation exercise with public health policy and practice stakeholders to identify further grey literature such as evaluations of relevant local interventions that were not in the public domain. Consultation with members of the public was conducted to identify search terms based on local intelligence. The lead author (VJM) attended a community learning event facilitated by a local community interest company and gave a short presentation on the study. Local residents attending the event were invited to complete a postcard listing factors they felt would have a positive or negative impact on health, wellbeing, and inequalities in the places they live. Their responses were collated and incorporated into our search terms (Additional file 1 lists their responses).

### Inclusion criteria

Following standard evidence synthesis approaches [58], the inclusion criteria for the review were developed in terms of PICOS (population, interventions, comparison, outcome, and setting) [39]. In keeping with criteria from the Database of Abstracts of Reviews of Effects (DARE), three key elements were required for systematic reviews to be included: 1) a clear question; 2) a transparent method for the search, selection and appraisal of evidence or studies; 3) a synthesis of results or evidence [59].

#### Population

Children and adults (all ages).

#### Intervention

Place-based interventions were eligible for inclusion if they focussed on one or more key elements of place and health: the physical, social, or economic environment. The inclusion criteria were purposely broad to allow for a wide range of different interventions to be located. For the purposes of the review, a place-based intervention was defined as any intervention, policy, programme, or action that aimed to improve health and reduce health inequalities that was delivered at a local- or regional-level, excluding national level interventions. Studies were excluded if they focused on interventions implemented before 2008 or were compositional interventions (individual behaviour change studies).

#### Comparison

We included systematic reviews that comprised studies with and without controls. Acceptable controls included randomised or matched designs.

#### Outcomes

Reviews measuring health (physical and mental, mortality) including health behaviours (physical activity, dietary behaviours, active travel), measures of personal or community wellbeing, or outcomes relating to the social determinants of health, including social cohesion, crime and safety, housing/neighbourhood condition and access to services, or training and employment opportunity outcomes were eligible for inclusion. Studies were excluded if they focused on the treatment of illnesses. Secondary outcomes included measures of inequalities in these health outcomes between groups or populations according to the PROGRESS+ factors (Table 1).

**Table 1:** PROGRESS+ Factors

PROGRESS+ Factors Adapted from Kavanagh et al. [58] and O’Neill et al. [60]	
**P**lace of residence	Rural/urban/inner-city, housing characteristics, social housing.
**R**ace, ethnicity, cultural background	Racial, ethnic, or socio-cultural background.
**O**ccupation	Employment status, type of occupation, employment-based benefits, unemployment, out-of-work benefits.
**G**ender and sex	Biological and gender-based differences and characteristics.
**R**eligion	Religious background/affiliation.
**E**ducation	Level of education attained and/or years spent in education, school type.
**S**ocial capital	Social relationships and networks, trust between community members, civic participation, collective community action, shared community goals.
**S**ocio-economic status	Income, welfare, assets/resources at individual or household level.
**+**	Age, disability, citizen status (e.g. refugee or displaced), minority groups. Instances where a person may be temporarily at a disadvantage; respite care or time in hospital. Instances where features of relationships place a person at a disadvantage; smoking parents or being excluded from school.

#### Setting

Only studies focusing on place-based interventions from high income countries (as defined by the World Bank list [61] at least once since 2008) were included.

### Study selection

Table 2 lists the inclusion and exclusion criteria used to assess identified studies.

**Table 2:** Inclusion/Exclusion Criteria

Inclusion	Exclusion
1. The publication is a systematic review, as defined in DARE criteria.	1. The publication is not a systematic review.
2. The publication includes interventions/policies in countries defined at least once since 2008 as a high-income country by the World Bank.	2. The publication was published, or intervention delivered, before 2008 or in a low-, lower-middle, or upper-middle income country.
3. The publication covers primary prevention local or regional public health interventions affecting change in one or more of the key elements of place as defined in PICOS.	3. The publication covers individual behaviour change, clinical, treatment or national-level interventions/policies.
4. The publication reports health (including health behaviours or factors associated with social determinants of health) or health inequalities outcomes in and between populations, disaggregated by one or more of the PROGRESS+ factors as defined in PICOS.	4. The publication does not include a relevant overall health outcome or disaggregated data by or between population groups.

### Screening and data extraction

All titles and abstracts were independently screened by two of four reviewers (VJM, RM, EM/SR, SB) using Rayyan QCRI (https://rayyan.qcri.org/welcome) [62] and relevant papers were retrieved and assessed for inclusion. Discrepancies were resolved by consensus. Full text screening was also conducted independently (VJM, SB and RM, SR) and discrepancies were resolved through discussion. Agreement between the reviewers was 95% (Cohen’s kappa: 0.89). After piloting the data extraction process (VJM), data extractions were conducted independently by two reviewers (VJM and SB) and then checked by the lead author (VJM). Data relating to the intervention, participants, outcomes, results, conclusions and researcher recommendations were extracted from identified systematic reviews. Key data (relating to PICOS) from full-text versions of included reviews were extracted using standard extraction forms [63] (See Additional file 1).

Data were only extracted from the systematic reviews and any relevant supplementary materials. We did not locate and extract data from the primary studies included in the identified reviews.

### Quality appraisal

Systematic review evidence was appraised independently (by VJM and SB), and then checked by the lead author with discrepancies resolved through discussion, using the revised Assessment of Multiple Systematic Reviews (R-AMSTAR), which provides a quantifiable assessment of systematic review quality [64]. The R-AMSTAR tool lists 11 questions to determine the quality of the review. Each question is answered yes or no based on assessing the review and then assigning a score of 1 to 4 based on a set of criteria in response to each question. For example, question 1 asks “was an “a priori” design provided?” if the review discusses an a priori design this question will be answered “yes” and then be graded by the following set of criteria “A: a clearly focused (PICO-based) question; B: Description of inclusion criteria; C: Study protocol is published and/or registered in advance. If the review meets all 3 of these criteria it will score 4; 2 criteria scores 3; 1 criteria scores 2; 0 criteria scores 1. Once all questions were completed the total score was calculated and in keeping with previous studies they were converted to low (11–22), medium (23–33), and high (34–44) quality ratings (see Table 3) [47, 64]. The quality of the reviews (as ascertained by VJM and SB) as well as the underpinning primary studies (as ascertained by the systematic review authors) are reported.

**Table 3:** Quality assessment for included reviews

KEY				1. Was an a priori design provided?		2. Was there duplicate study selection and data extraction?		3. Was a comprehensive literature search performed?		4. Was the status of publication used as an inclusion criteria?		5. Was a list of studies (included and excluded) provided?		6. Were the characteristics of the included studies provided?		7. Was the scientific quality of the included studies assessed and documented?		8. Was the scientific quality of the included studies used appropriately in formulating conclusions?		9. Were the methods used to combine the findings appropriate?		10. Was the likelihood of publication bias assessed?		11. Was the conflict of interest included?		R-AMSTAR SCORE
Y	YES	N	NO																							
Study																										
Brown et al (2015)				N	2	N	1	Y	4	Y	3	N	1	Y	4	Y	4	Y	2	Y	1	N	1	N	3	26 (medium)
Audrey & Batista-Ferrer (2015)				Y	3	Y	2	Y	4	Y	3	N	2	Y	2	Y	4	Y	4	Y	2	Y	2	N	1	31 (medium)
Hunter et al (2015)				N	2	N	1	Y	3	N	1	N	1	Y	3	Y	3	Y	4	Y	1	N	1	N	2	22 (low)
Mayne et al (2015)				N	2	N	1	N	1	Y	2	Y	1	Y	3	Y	4	Y	3	N	1	N	1	N	2	21 (low)
Sauni et al (2015)				Y	4	Y	4	Y	4	Y	4	Y	4	Y	4	Y	4	Y	4	Y	4	Y	2	N	3	41 (high)
McCartney et al (2017)				N	2	N	1	N	2	Y	2	N	1	Y	1	Y	1	Y	4	Y	1	N	1	N	1	17 (low)
Macmillan et al (2018)				N	3	Y	4	Y	4	Y	2	Y	4	Y	4	Y	4	Y	4	Y	1	N	1	N	3	34 (high)
Moore et al (2018)				Y	4	Y	4	Y	4	Y	3	Y	4	Y	4	Y	4	Y	2	Y	3	N	1	N	3	36 (high)
Stappers et al (2018)				Y	3	Y	3	Y	3	Y	2	Y	3	Y	3	Y	4	Y	3	N	1	N	1	N	2	28 (medium)
Tseng et al (2018)				Y	4	Y	4	Y	4	Y	3	N	1	Y	3	Y	4	Y	4	Y	2	Y	2	N	2	33 (medium)
Hunter et al (2019)				Y	4	Y	4	Y	4	Y	3	Y	1	Y	4	Y	4	Y	4	Y	3	N	2	N	3	36 (high)
Ige et al (2019)				N	2	Y	2	Y	4	Y	2	Y	1	N	1	Y	3	Y	4	N	1	N	1	N	2	23 (medium)
Persaud et al (2019)				Y	4	Y	4	Y	3	Y	3	Y	3	Y	4	Y	4	Y	4	Y	1	N	1	N	2	33 (medium)

### Synthesis

Systematic reviews meeting the inclusion criteria were tabulated and grouped by intervention type. Themes were identified from across studies of similar types of interventions and a narrative synthesis [65] was used to describe the learning from across the reviews.

## Results

**Fig. 1:**
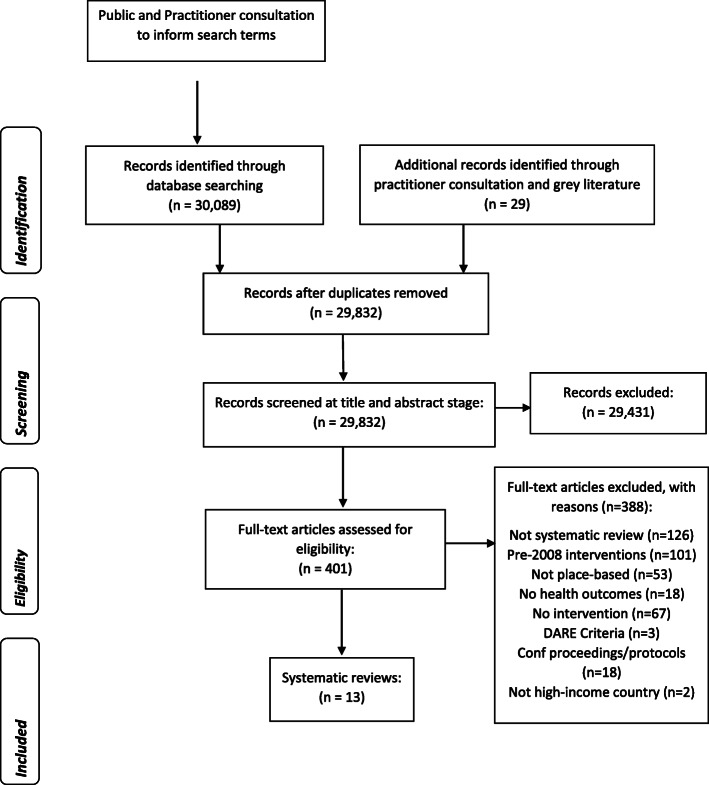
Adapted PRISMA Flow chart of selection procedure

A total of 30,089 citations were retrieved from the nine databases searched and an additional 29 records identified through the grey literature and stakeholder consultation exercises as part of the full project discussed in the methods section. All records were uploaded to Rayyan and after deduplication, a total of 29,832 unique citations were included in the title and abstract screening. The process of inclusion and exclusion is depicted in the PRISMA flow chart [42] in Fig. 1.

The reasons for exclusion at full-text review stage (*n* = 388) are available in Additional file 1. In total 13 systematic reviews were included in our review [21, 22, 66–76], reporting a total of 361 unique primary studies. Study characteristics reported in the systematic reviews were screened to ensure we only reported on interventions delivered since the 2008 economic crisis. This resulted in only 51 primary studies being eligible for data extraction from the systematic review and synthesis in this umbrella review. All but one study included in the reviews focused on interventions to change one or more element of the physical environment (*n* = 50); one study reported changes to the economic environment. No reviews meeting our inclusion criteria included studies that assessed the impact of social interventions on our outcomes of interest. Nine primary studies were included in more than one systematic review; to prevent duplication of their findings, data were only extracted from one review. As we only found 13 relevant systematic reviews, we have synthesised below the results of the underpinning relevant primary studies included within them rather than focusing exclusively on the overall findings of the reviews. This gives a fuller overview of the evidence base − whilst also ensuring that only those primary studies relevant to our research question are synthesised.

Within the included systematic reviews, 14 studies reported the effects of creating new, or renovating existing, infrastructure to increase physical activity (cycling/walking routes *n* = 11; bicycle share scheme *n* = 1; outdoor gyms *n* = 2); 16 studies reported the effects of home modifications (*n* = 3) of housing provision (*n* = 13); four studies reported greening/improvements to the public realm; six studies assessed changes to playgrounds/parks; four studies reported the opening or construction of new supermarkets; four studies reported transport interventions; two studies reported multi-component interventions (*n* = 1 housing refurbishments, improvements to the physical environment, external maintenance, community engagement, employment training, and creation of community space; *n* = 1 moving to a Smart Growth community with greater building density, less auto-dominated, greater non-residential land use, fewer barriers to connectivity, more parks and playgrounds, more traffic safety and aesthetic features, and fewer physical incivilities such as graffiti and litter); and one study reported the effects of congestion changes.

In terms of reported outcomes relating to these interventions, 27 studies assessed health behaviours (physical activity, diet, or active transport); 21 studies reported health outcomes using validated tools (e.g. EQ-5D Quality of Life tool); and three studies reported outcomes relating to the social determinants of health (social isolation, fear of crime, perception of area/safety, crime rates, and gun assaults). Only one review used PROGRESS+ factors (Table 1) to examine equity effects of the included primary studies [66], however, the authors concluded there was inadequate information to assess variation in outcomes. Across all included reviews, there was a lack of reporting on PROGRESS+ characteristics. Where studies did report these factors (such as area disadvantage, ethnicity, sex, income, or age), they were not used to assess variation between groups to identify any effect on health inequalities. Moreover, some factors were completely absent from the population characteristics included in the study summaries (occupation, religion, sexuality, gender minorities, and education).

Studies were located in the US (*n* = 24); Canada (*n* = 12); Australia (*n* = 7); UK (*n* = 6); New Zealand (*n* = 1); and Sweden (*n* = 1). Using the R-AMSTAR tool, reviews were graded as high (*n* = 4), medium (*n* = 6) or low (*n* = 3) quality (see Table 3). Studies from high and medium quality reviews are narratively synthesised below (with data extracted from the review) by intervention type and all reviews summarised in Table 4 (further details provided in Additional file 1).

**Table 4:** Summary of studies from included reviews

Review authors	Intervention(s) (n = no. of studies)	Outcome(s)	Summary results ↑ = increase/ improved ↓ = decrease/ deteriorated ↔ = no change (represent sig. Results unless reported otherwise)	R-AMSTAR quality appraisal rating
**Physical environment interventions (*n*** **= 50)**				
***Infrastructure to encourage physical activity (n = 14)***				
**Cycling and walking routes (*n*** **= 12)**				
Hunter et al. (2015) [70]	New greenway (*n* = 1)	Health behaviours: physical activity	↔	22 (low)
Mayne et al. (2015) [71]	Bike lane (*n* = 2); bike share (*n* = 1)	Health behaviours: physical activity, active transport.	Bike lane ↑; bike share ↑	21 (low)
Macmillan et al. (2018) [73]	Cycle lanes (*n* = 1)	Health behaviours: physical activity	↓	34 (high)
Stappers et al. (2018) [75]	New on- and off-road walking/cycling routes (*n* = 7)	Health behaviours: physical activity	Extended greenway ↔; cycling paths ↑; walking/cycling trail ↓; (greater ↑ for those in close proximity to intervention)	28 (medium)
**Outdoor gyms (*n*** **= 2)**				
Hunter et al. (2015) [70]	Outdoor gym (*n* = 1)	Health behaviours: Physical activity	Non-sig ↑	22 (low)
Hunter et al. (2019) [66]	Outdoor gym (*n* = 1)	Health behaviours: Physical activity	↑	36 (high)
***Housing (n = 16)***				
**Home modifications (*n*** **= 3)**				
Sauni et al. (2015) [72]	Home modifications (*n* = 1)	Health outcomes: asthma	↔	41 (high)
McCartney et al. (2017) [22]	Home modifications (*n* = 1)	Health outcomes: self-reported physical and mental health.	↑	17 (low)
Ige et al. (2019) [67]	Home modifications (*n* = 1)	Health outcomes: injuries from falls	↓ in injuries from falls (positive effect)	23 (medium)
**Provision of housing (*n*** **= 13)**				
Persaud et al. (2019) [68]	Provision of housing (*n* = 13)	Health outcomes: 34 reported including quality of life, physical health, mental health.	↑	33 (medium)
***Improving the public realm (n = 4)***				
Moore et al. (2018) [74]	Green storm water infrastructure; urban and landscape development (*n* = 2)	Health outcomes: quality of life Social determinants outcomes: perceptions of safety and social cohesion	Green storm water infrastructure ↔ (perceptions of place/safety, social cohesion); Urban and landscape development ↑ (quality of life)	36 (high)
Hunter et al. (2019) [66]	Greening vacant lots (*n* = 2)	Health outcomes: heart rate Social determinants outcomes: gun assaults, perceptions of safety, crime rates	↑ (perceptions of safety) ↓ (gun assaults, crime rates, and heart rate)	36 (high)
***Parks and playgrounds (n = 6)***				
Audrey & Batista-Ferrer (2015) [21]	Removal of park seating (*n* = 1)	Health behaviours: physical activity	↔	31 (medium)
Mayne et al. (2015) [71]	New park (*n* = 1)	Health behaviours: physical activity	↔	21 (low)
Hunter et al. (2015) [70]	Park re-development (*n* = 1)	Health behaviours: physical activity	↑	22 (low)
Hunter et al. (2019) [66]	Development of new, renovating existing, parks (*n* = 3)	Health behaviours: physical activity	↑	36 (high)
***Supermarkets (n = 4)***				
Macmillan et al. (2018) [73]	New supermarket/farmers market (*n* = 2)	Health outcomes: self-reported Body Mass Index (BMI). Health behaviours: physical activity; consumption of fruit and vegetables	New supermarket ↔ (self-report BMI); Farmers market ↑ (self-report fruit and vegetables); transport ↑ (physical activity for new users and greater for those living closer)	34 (high)
Tseng et al. (2018) [76]	New supermarkets (*n* = 2)	Health behaviours: consumption of fruit and vegetables Health outcomes: weight/BMI	Fruit and vegetable consumption ↔; overall caloric intake ↓; BMI↓ (intervention site); BMI↑ (control site)	33 (medium)
***Transport (n = 4)***				
Moore et al (2018) [74]	Segregated bus track with cycle/walking routes included (*n* = 1)	Health outcomes: mental health	↔ (when controlled from baseline)	36 (high)
Macmillan et al. (2018) [73]	New light rail transit (*n* = 3)	Health behaviours: physical activity.	↑ (for new users and greater for those living closer proximity); ↓ (for former users)	34 (high)
***Multi-component interventions (n = 2)***				
Audrey & Batista-Ferrer (2015) [21]	Relocation to Smart Growth Community (*n* = 1)	Health behaviours: physical activity	↔	31 (medium)
McCartney et al. (2017) [22]	Urban renewal programme (*n* = 1)	Health outcomes: self-reported health, perception of place.	↑ (perception of place) ↔ (self-reported health)	17 (low)
**Economic interventions (*n*** **= 1)**				
Brown et al. (2015) [69]	Traffic congestion charge (*n* = 1)	Health behaviours: active transport	↑	26 (medium)

### Physical environment interventions (*n* = 50)

The majority of studies (50/51) identified in the reviews reported on changes to the physical environment, either through the development of new infrastructure to provide opportunities for physical activity; modifying or providing housing; aesthetic improvements to the public realm; creating new supermarkets; developing existing, or creating new, transport facilities; or multi-component interventions that combine some, or all, of these elements plus others (for example employment training, creation of community spaces, and community engagement).

### Infrastructure to encourage physical activity (*n* = 14)

Five reviews [66, 70, 71, 73, 75] reported the findings of 14 studies relating to the development of new, or modification of existing, infrastructure to encourage physical activity. Twelve studies reported on the implementation of cycling and walking routes and two studies reported on the implementation of outdoor gyms.

#### Cycling and walking routes (*n* = 12)

Overall, reviews reported mixed results on physical activity outcomes pertaining to cycling and walking route interventions. Four reviews [70, 71, 73, 75] reported findings from 12 studies that examined physical activity outcomes after the introduction of cycling or walking routes. Six studies reported increases in physical activity and six reported no change or decreases in physical activity after the introduction of the interventions.

A medium quality review by Stappers et al. (2018) [75] reported on seven studies with critical to moderate risk of bias that assessed the effects of new on- and off-road walking and cycling routes on physical activity. Results were mixed, with four studies reporting no significant changes or negative effects on overall physical activity after the implementation of new cycling and walking routes, and three studies reporting an increase in cycling after construction of new separated bicycle paths. Three studies also assessed whether outcomes differed depending on proximity to the intervention; two found living closer to the intervention was associated with more cycling and walking, one found a higher increase in cycling for those living between 1.0–2.99 km from the intervention area compared with individuals living less than 1.0 km or further than 2.99 km away.

A high quality review by Macmillan et al. (2018) [73] reported one study that assessed changes in cycling behaviours after the installation of bicycle boulevards in local streets. The study was graded five out of nine for risk of bias and found that living in streets where the intervention was implemented was not associated with increased cycling.

#### Outdoor gyms (*n* = 2)

Two reviews [66, 70] reported findings from two studies assessing the impact of outdoor gyms on physical activity. Overall, evidence suggests small or non-significant increases in physical activity after the introduction of outdoor gyms.

High quality review evidence by Hunter et al. (2019) [66] reported findings from a moderate quality study assessing physical activity outcomes after the installation of an outdoor gym plus targeted promotional marketing and exercise sessions with professional trainers. Results showed a small but significant increase in senior park users engaging in moderate to vigorous physical activity at follow up.

### Housing (*n* = 16)

Four reviews [22, 67, 68, 72] reported the findings of 16 studies that related to the provision, or modification, of housing. Three studies study reported on the effects of home modifications and 13 studies reported on the provision of housing.

#### Home modifications (*n* = 3)

One high quality review by Sauni et al. (2015) [72] investigated the impact of home modifications on asthma-related health outcomes. Only one study in the review was implemented after 2008 and was graded as poor quality by the review authors. The study found no differences in respiratory outcomes for people living in homes that were undergoing repair after a flood compared to those living in homes that had already been repaired. One high quality study in a medium quality review by Ige et al. (2019) [67] reported on injury prevalence after the implementation of a home modification intervention. Findings showed a 26% reduction in the rate of home injuries caused by falls in the groups that received home modifications.

#### Provision of housing (*n* = 13)

One medium quality review by Persaud et al. (2019) [68] explored ‘Housing First’ interventions which combined the provision of housing with treatment for various addictions, mental health challenges, and other social supports. Thirteen studies in the review were implemented after 2008 and were graded as medium to high quality by the review authors. Studies reported a total of 34 different health outcomes, of which 31 were statistically significant, and 3 had unknown significance. Of the 31 statistically significant outcomes, 12 outcomes (from 12 studies) favoured the intervention, and 19 outcomes (from 11 studies) favoured the control. Although this presents mixed results, all control groups had treatment for addiction, mental health challenges, and other social supports, but limited detail was provided on these to contextualise the findings. Duration of studies varied (from six to 180 months) and this may provide insight into the differing outcomes for intervention and control groups.

### Improving the public realm (*n* = 4)

Two high quality reviews [66, 74] reported the findings from four studies that assessed the effects of improvements to the public realm on health and outcomes relating to the social determinants of health. One study, graded as moderate risk of bias by Moore et al. (2018) [74], found that landscape changes to improve watershed function and stormwater capacity by planting trees had no effect on fear of crime or whether people felt the neighbourhood was friendly or sociable compared to a control site. A second study identified by Moore et al. (2018) [74] graded as serious risk of bias, assessed changes in quality of life after streets were redesigned to look more attractive and safer with buildouts to slow traffic, planters, benches, and light. Quality of life for respondents living in interventions streets improved compared to a decrease for respondents living in control streets. Hunter et al. (2019) [66] reported findings from two studies assessing how improvements to vacant lots affect health and social determinants of health outcomes. One high quality study (quality assessment score 11/11) examined the greening of vacant lots, which included removing debris, planting grass and trees, and erecting a wooden fence. Compared to a control lot with no greening intervention, there was a non-significant decrease in the number of total crimes and gun assaults, and people reported feeling significantly safer around greened vacant lots.

A second similar study of moderate quality (score 7/11), to improve vacant lots found that when participants were in view of the intervention sites, compared to controls, this led to a significant reduction in heart rate for African Americans.

### Parks and playgrounds (*n* = 6)

Overall, four reviews [21, 66, 70, 71] reported the results from six studies that showed significant increases in physical activity after the introduction of new, or renovation of existing, parks or playgrounds. Two reviews [21, 71] reported no differences in physical activity from two studies after the introduction of a new park and the removal of seating in an existing park.

A high quality review by Hunter et al. (2019) [66] reported three studies assessing the effect of implementing new, or renovation of existing, parks. One study, graded nine out of 11 for quality, evaluated the development of a new park and found a significant increase in the proportion of park users engaging in moderate or vigorous physical activity. Another study, graded nine out of 11 for quality, examined the impact of replacing old playground equipment and ground surfacing compared to control parks with no renovations and found significant increases between baseline and 12-month follow up for the number of people engaged in moderate to vigorous physical activity compared with the control park. The third study was graded 11 out of 11 for quality and examined soft measures in park renovations (new walking path signs), promotional incentives (water bottles, park-branded key chains, targeted emails), and outreach activities (hiring community engagement officers, buying activity materials). Results showed increased physical activity in intervention parks compared to declines in control parks.

Medium quality review evidence by Audrey and Batista-Ferrer (2015) [21] reported results from one low quality study that found removing seating arrangements in parks did not change the likelihood of children standing or engaging in moderate to physical activity.

### Supermarkets (*n* = 4)

Two reviews [73, 76] reported findings from four studies that assessed changes in health behaviours and health outcomes after the development of new supermarkets. A high quality review by MacMillan et al. (2018) [73] reported two studies that assessed changes in self-reported body mass index (BMI) and purchases and consumption of fresh fruit and vegetables after the introduction of a new supermarket (risk of bias 5/9) and a weekly farmers market (risk of bias 2/9). There were no significant changes in self-reported BMI after the introduction of the new supermarket, however, there were self-reported increases in fruit and vegetable consumption associated with the farmer’s market. A medium quality review by Tseng et al. (2018) [76] reported two studies that assessed the impact of two new supermarkets on BMI and caloric intake. One low risk of bias study reported no difference in fruit and vegetable consumption, but an overall decline in caloric intake and a non-significant reduction in BMI for the intervention neighbourhood. A high risk of bias study also reported reductions in BMI after the opening of a new grocery store in the centre of a neighbourhood that had no other food stores within walking distance, whereas the control town reported increases in BMI.

### Transport (*n* = 4)

Two high quality reviews [73, 74] reported findings from 4 studies that assessed the impact of new transport facilities on health behaviours and health outcomes. MacMillan et al. (2018) [73] reported findings from 3 studies assessing the impact of new light rail transit on physical activity. One study (risk of bias 4/9) found a negative association between total walk trips at follow up based on the interaction of the distance to the rail stop group and baseline walking trips after the opening of a new light rail transit line. Two studies reported findings from the same intervention (risk of bias for both 6/9) that extended a light rail line and found increased physical activity among new line users, however, former users experienced decreased physical activity. Moreover, there were greater increases in physical activity for those living in closer proximity to the intervention. Moore et al. (2018) [74] reported findings from one study (no quality assessment or risk of bias) that assessed the impact on mental health outcomes after the development of a new purpose-built guided segregated bus track with cycle and walking routes included. Although mental health outcomes improved for respondents who used the new route for active transport, this effect attenuated when controlled from baseline mental health.

### Multi-component interventions (*n* = 2)

Two reviews [21, 22] reported two studies that assessed changes in health behaviours and health outcomes after the implementation of interventions comprising multiple elements. One medium quality review by Audrey and Batista-Ferrer (2015) [21] reported findings from one moderate quality study that assessed changes in physical activity in children and young people after moving to a Smart Growth community; which is characterised as having greater building density, less auto-dominated form, greater non-residential land uses, fewer barriers to connectivity, more parks and playgrounds, more traffic safety and aesthetic features, and fewer physical incivilities such as graffiti and litter. Compared to a control site, the study reported no strong evidence for increases in moderate to vigorous activity in the Smart Growth group.

### Economic interventions (*n* = 1)

Only one review reported on changes to the economic environment, focussing on traffic congestion pricing schemes. A medium quality review by Brown et al. (2015) [69] examined changes in health behaviours after the introduction of traffic congestion charges. Only one moderate quality study reported findings from a scheme implemented after 2008. The study findings suggests that traffic congestion pricing schemes lead to increases in health-related behaviours such as shifting from car trips to public transport. The study reported a 24% increase in the total number of trips by public transport, 9% decrease in commuter car trips, a decrease in all discretionary trips, and a 36% decrease in community cycling trips after traffic congestion charges were introduced. However, the study authors noted these findings were unreliable due to adverse weather conditions and a small and unrepresentative sample.

### Health inequalities

Only one review used the PROGRESS+ tool to examine the equity effects of interventions reported in the primary studies. However, Hunter et al. (2019) [66] reported there was insufficient evidence relating to the equity effects of urban green space interventions to draw firm conclusions. Although they reported some primary studies were located in deprived neighbourhoods and did show positive effects, the evidence was generally mixed, and none of these studies met our inclusion criteria. The studies included in our review from Hunter et al. (2019) [66] all lacked sufficient information on PROGRESS+ factors to assess any effects on health inequalities.

## Discussion

Place-based inequalities in health are persistent. This umbrella review has examined place-based interventions that aimed to alter the environments in which people live. Thirteen systematic reviews were included in this umbrella review, comprising 51 unique primary studies. The majority of these studies (*n* = 50) focused on changes to the physical environment, one assessed changes to the economic environment, and none reported on interventions pertaining to the social environment that includes our outcomes of interest.

Given the considerable heterogeneity – and quality - both across the 13 systematic reviews and the 51 studies included therein, a cautious assessment is made of the overall effect on health outcomes, or more specifically health behaviours due to most outcomes focusing on physical activity. There is tentative evidence that the provision of housing/home modifications, improving the public realm, parks and playgrounds, supermarkets, transport, cycle lanes, walking routes, and outdoor gyms – can all have positive impacts on health outcomes – particularly physical activity. Moreover, the lack of reporting on PROGRESS+ factors constrains the conclusions that can be drawn about the effect on health inequalities. No studies assessed differences in outcomes based on PROGRESS+ factors, so the effect of these interventions on health inequalities remains unclear, a finding consistent with previous systematic review evidence [56]. Although this could also reflect systematic reviews failing to extract PROGRESS+ factors and effects on health inequalities from the primary studies.

When examining the reviews as a whole, the evidence suggests that some place-based approaches that change the physical environment can be effective at improving health, health behaviours, and social determinants of health outcomes with 37 studies (from 11 reviews [22, 66–71, 73–76]) reporting positive results. Although the focus of this review was to identify interventions that alter places rather than people, eight of the studies (from four reviews [66, 70, 71, 75]) reporting positive outcomes could be described as demonstrating lifestyle drift, where interventions change place but require individuals to alter their behaviour in order to achieve improvements in health outcomes [77].

Although these kind of physical changes to place appear to offer a more universal intervention approach that has potential to affect whole populations of people living in places [78], there is increasing evidence to suggest these types of interventions may result in widening of inequalities between people from different social groups [17, 18, 79–81]. Indeed, proximity to the intervention was shown to be associated with greater positive outcomes in six studies that directly assessed for this interaction which indicates that some members of this population may have benefitted disproportionately to others.

This could suggest that to address inequalities, emphasis should be placed on reducing the social gradient in health by adopting proportionate universalism whereby changes are made at a scale commensurate with the level of disadvantage, rather than focussing solely on areas of greatest need [34, 82]. However, there is also a need to consider the type of intervention delivered in these areas. There is evidence that suggests universal interventions that require individuals to use less agency can be more effective and reduce health inequalities, particularly in comparison to those that require high levels of individual choice [19]. Thus indicating that interventions succumbing to lifestyle drift, changing the physical place but requiring individuals to choose to engage in the intervention [18, 80] (e.g. creating cycle paths or spaces to engage in physical activity), may be less effective and unlikely to reduce inequalities than those that require less change at the individual level (e.g. creating inclusive and sustainable social and economic environments [34]).

Indeed, 29 of the studies that reported positive outcomes (from nine reviews [22, 66–70, 73, 76]) examined low agentic interventions; provision of housing/home modifications, improving the public realm, parks and playgrounds, supermarkets, transport, multi-component and economic interventions. In contrast, eight studies (from four reviews [66, 70, 71, 75]) reporting positive results examined high agentic interventions; cycle lanes, walking routes, and outdoor gyms. However, presenting low and high agency interventions as a dichotomy and drawing conclusions that state one approach as more or less effective in terms of health outcomes or behaviours is perhaps misleading. Interventions deemed here as highly agentic, while they do require individuals to engage with the intervention to be beneficial in terms of health behaviours, may also have positive impacts that have not been captured. Indeed, new cycle paths and walking routes or installation of outdoor gyms could also be classified as improving the public realm which is associated with improved outcomes in terms of physical health, quality of life, perceptions of safety, and reduced crime rates [66, 74].

While evidence suggests level of agency is important in terms of effectiveness on health and health inequalities [19], there is also perhaps a need to widen the outcomes measured in place-based evaluations to capture the nuance between high and low agency interventions. For example, a systems approach to public health evaluations that captures broader outcomes on causal pathways to health outcomes may be more suitable for assessing the effectiveness of place-based interventions [83, 84]. Moreover, further work is required to consider ‘scaling up’ [28] and examining upstream policy changes, at international and national levels, that shape people and places as these affect what action can be taken at the local and regional level, for example housing legislation or provision of health and social care services. There are also notable gaps in the literature that we reviewed, particularly around the impact of economic interventions, cost effectiveness, and health inequalities. Indeed, these omissions prevent the ability to draw conclusions about what interventions are effective at improving health and reducing inequalities during austere times. Although there is evidence to suggest reductions in local government budgets in England are associated with widening inequalities in health [85], this review has been unable to identify whether the specific interventions reviewed here have been affected by austerity measures. This suggests a need for increased research in this area, although it may be the case that such studies have simply not yet been reviewed - in which case it is a gap for future systematic reviews to address.

### Implications for research and practice

There is ongoing interest in both public health practice and research to identify and implement effective and cost-effective place-based interventions to improve health and reduce inequalities. This umbrella review has provided a synthesis of recent evidence relating to the effectiveness of physical and economic environment changes that improve health outcomes, health behaviours, and the social determinants of health. It has found tentative evidence that the provision of housing/home modifications, improving the public realm, parks and playgrounds, supermarkets, transport, cycle lanes, walking routes, and outdoor gyms – can all have positive impacts on health outcomes – particularly physical activity. It has also highlighted there is an urgent need in both research and practice to capture PROGRESS+ data in order to assess variation in outcomes across multiple factors. Currently the effects of these interventions on health inequalities are unknown due to a lack of clear reporting at the systematic review level, or collection of sufficient data at the primary study level. Additionally, there is a need to measure broader outcomes associated with the social determinants of health, such as perceptions of place, beyond health behaviours as interventions may be having positive effects that are unknown. This could be facilitated by working with those living in the areas of greatest need to understand what elements of the environment need to change and what outcomes should be measured.

### Strengths and limitations

This umbrella review presents several strengths. Our methodological approach included a broad and wide-ranging search that included both database and grey literature searches. In addition, we conducted a consultation exercise with public health policy and practice stakeholders to identify evaluations that were not in the public domain, as well as consultations with members of the public to identify search terms that they felt represented factors in their communities that improve health and wellbeing. However, there were also limitations. Our review excluded systematic reviews and any primary studies contained therein that reported on interventions delivered pre-2008. Although this was to address our central aim to demonstrate what place-based interventions are effective in austere times, and update previous evidence on this topic, this will have restricted the evidence base significantly. Indeed, some of the systematic reviews included important studies on place-based urban regeneration schemes that were implemented before 2008 (such as GoWell [86] and Moving to Opportunity [87]) and, as such, were excluded from this review. Moreover, a focus on local place-based strategies to improve health also fails to capture how local policy options are constrained by wider social, political, and economic factors that operate at regional, national, and global levels [2]. Furthermore, distinguishing between place-based and individual based interventions during the screening phase of this review was not without challenges, given many place-based interventions are affected by lifestyle drift. Indeed, some place-based interventions included in this review that changed physical aspects of the environment could be defined as demonstrating lifestyle drift as they require high levels of individual agency to achieve any health benefits [19, 77]. However, only reviews that could demonstrate change in the physical, economic, or social environments were included, while reviews that focused on the individual (e.g., physical activity, weight management, or dietary interventions) were excluded. A further limitation arises due to much of the evidence identified in this umbrella review originating from North America, as is commonly the case in systematic reviews, which could mean the generalisability of the findings to other high-income countries may be restricted. Relatedly, it is unclear to what extent, if any, all local governments were required to reduce their budgets as a result of the global financial crisis of 2008 and, as such, the evidence presented here may not reflect changes to the wider economic environment. However, there is emerging evidence to suggest reductions in local government funding in England are associated with widening inequalities in health [85]. There is also a high possibility of publication bias, in that negative results are less likely to be published within the primary studies. Indeed, many of the systematic reviews we assessed scored low for assessing publication bias within their included primary studies. Umbrella reviews may also be restricted in their ability to provide robust conclusions due to their reliance on information extracted at the systematic review level. Primary studies may include more information than is extracted by systematic reviewers, for example including sub-group analysis of PROGRESS+ factors to examine inequalities. Moreover, the systematic reviews may misreport information extracted from primary studies [88].

## Conclusion

Some place-based interventions that make the physical environment more salutogenic can be effective at improving physical health, health behaviours and social determinants of health outcomes. High agentic interventions indicate greater improvements for those living in greater proximity to the intervention, which may suggest that in order for interventions to reduce inequalities, they should be implemented at a scale commensurate with the level of disadvantage. Future research needs to ensure equity data is collected, as this is severely lacking and impeding progress on identifying interventions that are effective in reducing health inequalities.

## Supplementary Information

**Additional file 1.**

## Data Availability

All data generated or analysed during this study are included in this published article (and its supplementary files). Extraction forms for all included systematic reviews are available from the corresponding author on reasonable request.

## References

[1] Arcaya MC, Tucker-Seeley RD, Kim R, Schnake-Mahl A, So M, Subramanian SV (2016). Research on neighborhood effects on health in the United States: a systematic review of study characteristics. Soc Sci Med.

[2] Bambra C (2016). Health Divides: Where You Live Can Kill You.

[3] Office of National Statistics (2018). Health State Life Expectancies, UK: 2015 to 2017.

[4] González S (2010). The north/south divide in Italy and England: discursive construction of regional inequality. Eur Urban Reg Stud.

[5] Gutiérrez-Fisac JL, Gispert R, Solà J (2000). Factors explaining the geographical differences in disability free life expectancy in Spain. J Epidemiol Commun Health.

[6] Thomson KH, Renneberg AC, McNamara CL, Akhter N, Reibling N, Bambra C (2017). Regional inequalities in self-reported conditions and non-communicable diseases in European countries: findings from the European social survey (2014) special module on the social determinants of health. Eur J Pub Health.

[7] Larsen K. The health impacts of place-based interventions in areas of concentrated disadvantaged: a review of the literature. Liverpool: Sydney South West Area Health Service; 2007. https://www.swslhd.health.nsw.gov.au/populationhealth/PH_environments/pdf/Rpt_Intervention_Impact.pdf.

[8] Cummins S, Curtis S, Diez-Roux AV, Macintyre S (2007). Understanding and representing “place” in health research: a relational approach. Soc Sci Med.

[9] Evans TG, Evans RG, Barer ML, Marmor TR (2007). Why are some people healthy and others not? The determinants of health of populations. Health Hum Rights.

[10] Macintyre S, Ellaway A, Cummins S. Place effects on health: how can we conceptualise, operationalise and measure them? Soc Sci Med. 2002. 10.1016/S0277-9536(01)00214-3. 10.1016/s0277-9536(01)00214-3 12137182

[11] Macintyre S (2007). Deprivation amplification revisited; or, is it always true that poorer places have poorer access to resources for healthy diets and physical activity?. Int J Behav Nutr Phys Act.

[12] KANER EFS, DICKINSON HO, BEYER F (2009). The effectiveness of brief alcohol interventions in primary care settings: a systematic review. Drug Alcohol Rev.

[13] TAIT RJ, HULSE GK (2003). A systematic review of the effectiveness of brief interventions with substance using adolescents by type of drug. Drug Alcohol Rev.

[14] Ma C, Avenell A, Bolland M, Hudson J, Stewart F, Robertson C, Sharma P, Fraser C, MacLennan G (2017). Effects of weight loss interventions for adults who are obese on mortality, cardiovascular disease, and cancer: systematic review and meta-analysis. BMJ.

[15] Brown T, Summerbell C (2009). Systematic review of school-based interventions that focus on changing dietary intake and physical activity levels to prevent childhood obesity: an update to the obesity guidance produced by the National Institute for health and clinical excellence. Obes Rev.

[16] Bambra CL, Hillier FC, Cairns J-M, Kasim A, Moore HJ, Summerbell CD (2015). How effective are interventions at reducing socioeconomic inequalities in obesity among children and adults? Two systematic reviews. Public Health Res.

[17] Hillier-Brown FC, Bambra CL, Cairns JM, Kasim A, Moore HJ, Summerbell CD (2014). A systematic review of the effectiveness of individual, community and societal-level interventions at reducing socio-economic inequalities in obesity among adults. Int J Obes.

[18] McLaren L, McIntyre L, Kirkpatrick S (2010). Rose’s population strategy of prevention need not increase social inequalities in health. Int J Epidemiol.

[19] Adams J, Mytton O, White M, Monsivais P (2016). Why are some population interventions for diet and obesity more equitable and effective than others? The role of individual agency. PLoS Med.

[20] O’Dwyer LA, Baum F, Kavanagh A, MacDougall C (2007). Do area-based interventions to reduce health inequalities work? A systematic review of evidence. Crit Public Health.

[21] Audrey S, Batista-Ferrer H (2015). Healthy urban environments for children and young people: a systematic review of intervention studies. Health Place.

[22] McCartney G, Hearty W, Taulbut M, Mitchell R, Dryden R, Collins C (2017). Regeneration and health: a structured, rapid literature review. Public Health.

[23] Gibson M, Petticrew M, Bambra C, Sowden AJ, Wright KE, Whitehead M (2011). Housing and health inequalities: a synthesis of systematic reviews of interventions aimed at different pathways linking housing and health. Health Place.

[24] Macintyre S, Maciver S, Sooman A (2009). Area, class and health: should we be focusing on places or people?. J Soc Policy.

[25] John P, Ward H (2005). How competitive is competitive bidding? The case of the single regeneration budget program. J Public Adm Res Theory.

[26] John P, Ward H, Dowding K (2004). The bidding game: competitive funding regimes and the political targeting of urban programme schemes. Br J Polit Sci.

[27] Taylor P, Turok I, Hastings A (2001). Competitive bidding in urban regeneration: stimulus or disillusionment for the losers?. Environ Plan C Gov Policy.

[28] Bambra C, Smith KE, Pearce J (2019). Scaling up: The politics of health and place. Soc Sci Med.

[29] Naik Y, Baker P, Ismail SA, Tillmann T, Bash K, Quantz D, Hillier-Brown F, Jayatunga W, Kelly G, Black M, Gopfert A, Roderick P, Barr B, Bambra C (2019). Going upstream - an umbrella review of the macroeconomic determinants of health and health inequalities. BMC Public Health.

[30] Halliday E, Brennan L, Bambra C, Popay J (2021). ‘It is surprising how much nonsense you hear’: how residents experience and react to living in a stigmatised place. A narrative synthesis of the qualitative evidence. Health Place.

[31] Bambra C (2019). Health in Hard Times.

[32] Mattheys K, Bambra C, Warren J, Kasim A, Akhter N (2016). Inequalities in mental health and well-being in a time of austerity: baseline findings from the Stockton-on-Tees cohort study. SSM Popul Healtht.

[33] Bambra C, Riordan R, Ford J, Matthews F. The COVID-19 pandemic and health inequalities. J Epidemiol Community Health. 2020:jech-2020-214401. 10.1136/jech-2020-214401. 10.1136/jech-2020-214401 PMC7298201 32535550

[34] Public Health England (2021). Inclusive and Sustainable Economies: Leaving No One behind. Supporting Place-Based Action to Reduce Health Inequalities and Build Back Better.

[35] Masters R, Anwar E, Collins B, Cookson R, Capewell S (2017). Return on investment of public health interventions: a systematic review. J Epidemiol Community Health.

[36] Barr B, Bambra C, Whitehead M, Duncan WH. The impact of NHS resource allocation policy on health inequalities in England 2001-11: longitudinal ecological study. BMJ. 2014;348(may27 6). 10.1136/bmj.g3231. 10.1136/bmj.g3231 PMC4035504 24865459

[37] Bambra C, Munford L, Brown H (2018). Health for Wealth: Building a Healthier Northern Powerhouse for UK Productivity.

[38] McGowan VJ, Mead R, McGill E (2019). Examining the effects, and cost-effectiveness, of place-centred strategies to improve health and reduce inequalities: a systematic review protocol.

[39] Higgins JPT, Thomas J, Chandler J (2019). Cochrane Handbook for Systematic Reviews of Interventions.

[40] Rychetnik L, Frommer M. A Schema for evaluating evidence on public health interventions; version 4. Melbourne; 2002. 10.1136/jech.56.2.119 PMC1732065 11812811

[41] Khan KS, Ter Riet G, Glanville J, Sowden AJ, Kleijnen J (2001). Undertaking systematic reviews of research on effectiveness CRD’s guidance for those carrying out or commissioning reviews. CRD Report Number 4.

[42] Liberati A, Altman DG, Tetzlaff J, et al. The PRISMA statement for reporting systematic reviews and meta-analyses of studies that evaluate health care interventions: explanation and elaboration. J Clin Epidemiol. 2009. 10.1016/j.jclinepi.2009.06.006. 10.1016/j.jclinepi.2009.06.006 19631507

[43] Bambra C, Gibson M. Case study of public health. In: Biondi-Zoccai G, editor. Umbrella Reviews - Evidence Synthesis with Overviews of Reviews and Meta-Epidemiologic Studies. Switzerland: Springer; 2016.

[44] McMahon N, Thomson K, Kaner E, Bambra C (2019). Effects of prevention and harm reduction interventions on gambling behaviours and gambling related harm: an umbrella review. Addict Behav.

[45] Thomson K, Hillier-Brown F, Todd A, McNamara C, Huijits T, Bambra C. The effects of public health policies on health inequalities: A review of reviews. Lancet. 2017;390:S12. 10.1186/s12889-018-5677-1 PMC6044092 30005611

[46] Cairns J, Warren J, Garthwaite K, Greig G, Bambra C (2015). Go slow: An umbrella review of the effects of 20 mph zones and limits on health and health inequalities. J Public Health.

[47] Thomson K, Hillier-Brown F, Todd A, McNamara C, Huijts T, Bambra C (2018). The effects of public health policies on health inequalities in high-income countries: an umbrella review. BMC Public Health.

[48] Cochrane Effective Practice and Organisation of Care (2019). LMIC Filters.

[49] Bitton A, Fifield J, Ratcliffe H, Karlage A, Wang H, Veillard JH, et al. Primary healthcare system performance in low-income and middle-income countries: a scoping review of the evidence from 2010 to 2017. BMJ Glob Health. 2019;4(Suppl 8). 10.1136/bmjgh-2019-001551. 10.1136/bmjgh-2019-001551 PMC6703296 31478028

[50] Burchett HED, Kneale D, Blanchard L, Thomas J (2020). When assessing generalisability, focusing on differences in population or setting alone is insufficient. Trials.

[51] Harris T, Hodge L, Phillips D (2019). English Local Government Funding: Trends and Challenges in 2019 and Beyond.

[52] Stuckler D, Basu S, Suhrcke M, Coutts A, McKee M (2009). The public health effect of economic crises and alternative policy responses in Europe: an empirical analysis. Lancet.

[53] Taylor-Robinson D, Gosling R (2011). Local authority budget cuts and health inequalities. BMJ..

[54] Gray M, Barford A (2018). The depths of the cuts: the uneven geography of local government austerity. Cambridge J Reg Econ Soc.

[55] Beatty C, Fothergill S (2019). The Uneven Impact of Welfare Reform: The Financial Losses to Places and People.

[56] Bambra C, Gibson M, Sowden A, Wright K, Whitehead M, Petticrew M (2010). Tackling the wider social determinants of health and health inequalities: evidence from systematic reviews. J Epidemiol Community Health.

[57] Larsen K. The health impacts of place-based interventions in areas of concentrated disadvantaged: a review of the literature. Sydney; 2007. http://hiaconnect.edu.au/old/files/Place_Based_Interventions.pdf

[58] Kavanagh J, Oliver S, Lorenc T (2008). Reflections on developing and using PROGRESS-Plus Equity Update.

[59] Centre for Reviews and Dissemination UOY. The Database of Abstracts of Reviews of Effects (DARE). Effectiveness Matters. 2002;6(2):1-4.

[60] O’Neill J, Tabish H, Welch V (2014). Applying an equity lens to interventions: using PROGRESS ensures consideration of socially stratifying factors to illuminate inequities in health. J Clin Epidemiol.

[61] The World Bank (2020). The World Bank: High Income Countries.

[62] Ouzzani M, Hammady H, Fedorowicz Z, Elmagarmid A (2016). Rayyan-a web and mobile app for systematic reviews. Syst Rev.

[63] Higgins JP, Green S (2008). Cochrane Handbook for Systematic Reviews of Interventions: Cochrane Book Series.

[64] Kung J, Chiappelli F, Cajulis OO, Avezova R, Kossan G, Chew L, Maida CA (2010). From systematic reviews to clinical recommendations for evidence-based health care: validation of revised assessment of multiple systematic reviews (R-AMSTAR) for grading of clinical relevance. Open Dent J.

[65] Popay J, Roberts H, Sowden A (2006). Guidance on the conduct of narrative synthesis in systematic reviews. Prod ESRC Methods Program Version.

[66] Hunter RF, Cleland C, Cleary A, Droomers M, Wheeler BW, Sinnett D, Nieuwenhuijsen MJ, Braubach M (2019). Environmental, health, wellbeing, social and equity effects of urban green space interventions: a meta-narrative evidence synthesis. Environ Int.

[67] Ige J, Pilkington P, Orme J, et al. The relationship between buildings and health: a systematic review. J Public Health. 2019;41(2):e121–32. 10.1093/pubmed/fdy138 PMC6645246 30137569

[68] Persaud N, Steiner L, Woods H, et al. Health outcomes related to the provision of free, tangible goods: A systematic review. PLoS ONE. 2019;14(3):e0213845. 10.1371/journal.pone.0213845 PMC6426236 30893372

[69] Brown V, Moodie M, Carter R. Congestion pricing and active transport - evidence from five opportunities for natural experiment. J Transp Health. 2015;2(4):568–79. 10.1016/j.jth.2015.08.002.

[70] Hunter RF, Christian H, Veitch J, Astell-Burt T, Hipp JA, Schipperijn J. The impact of interventions to promote physical activity in urban green space: a systematic review and recommendations for future research. Soc Sci Med. 2015;124:246–56. 10.1016/j.socscimed.2014.11.051. 10.1016/j.socscimed.2014.11.051 25462429

[71] Mayne SL, Auchincloss AH, Michael YL. Impact of policy and built environment changes on obesity-related outcomes: a systematic review of naturally occurring experiments. Obes Rev. 2015;16(5):362–75. 10.1111/obr.12269. 10.1111/obr.12269 PMC4789114 25753170

[72] Sauni R, Verbeek JH, Uitti J, Jauhiainen M, Kreiss K, Sigsgaard T (2013). Remediating buildings damaged by dampness and mould for preventing or reducing respiratory tract symptoms, infections and asthma. Cochrane Database Syst Rev.

[73] MacMillan F, George ES, Feng X, et al. Do natural experiments of changes in neighborhood built environment impact physical activity and diet? a systematic review. Int J Environ Res Public Health. 2018;15(2):26. 10.3390/ijerph15020217 PMC5858286 29373567

[74] Moore THM, Kesten JM, López-López JA, Ijaz S, McAleenan A, Richards A, et al. The effects of changes to the built environment on the mental health and well-being of adults: systematic review. Health Place. 2018;53:237. 10.1016/j.healthplace.2018.07.012 30196042

[75] Stappers NE, Van Kann DH, Ettema D, De Vries NK, Kremers SP. The effect of infrastructural changes in the built environment on physical activity, active transportation and sedentary behavior - A systematic review. Health Place. 2018;53:135–49. 10.1016/j.healthplace.2018.08.002 30138827

[76] Tseng E, Zhang A, Shogbesan O, Gudzune KA, Wilson RF, Kharrazi H, et al. Effectiveness of policies and programs to combat adult obesity: a systematic review. J Gen Intern Med. 2018;33(11):1990–2001. 10.1007/s11606-018-4619-z PMC6206360 30206789

[77] Popay J, Whitehead M, Hunter DJ (2010). Injustice is killing people on a large scale—but what is to be done about it?. J Public Health.

[78] Kavanagh J, Oliver S, Lorenc T, Caird J, Tucker H, Harden A, Greaves A, Thomas J, Oakley A (2009). School-based cognitive-behavioural interventions: a systematic review of effects and inequalities. Health Soc Rev.

[79] Lehne G, Bolte G (2017). Impact of universal interventions on social inequalities in physical activity among older adults: an equity-focused systematic review. Int J Behav Nutr Phys Act.

[80] Frohlich KL, Potvin L (2008). Transcending the known in public health practice. Am J Public Health.

[81] Lorenc T, Petticrew M, Welch V, Tugwell P. What types of interventions generate inequalities? Evidence from systematic reviews. J Epidemiol Commun Health. 2013;67(2):190–3. 10.1136/jech-2012-201257. 10.1136/jech-2012-201257 22875078

[82] Egan M, Kearns A, Katikireddi SV, Curl A, Lawson K, Tannahill C. Proportionate universalism in practice? A quasi-experimental study (GoWell) of a UK neighbourhood renewal programme’s impact on health inequalities. Soc Sci Med. 2016;152:41–9. 10.1016/j.socscimed.2016.01.026. 10.1016/j.socscimed.2016.01.026 26829008

[83] Egan M, McGill E, Penney T, et al. NIHR SPHR Guidance on Systems Approaches to Local Public Health Evaluation. In: Part 2: what to consider when planning a systems evaluation. London; 2019. https://sphr.nihr.ac.uk/research/developing-a-syst.

[84] Rutter H, Savona N, Glonti K, Bibby J, Cummins S, Finegood DT, Greaves F, Harper L, Hawe P, Moore L, Petticrew M, Rehfuess E, Shiell A, Thomas J, White M (2017). The need for a complex systems model of evidence for public health. Lancet..

[85] Alexiou A, Fahy K, Mason K, Bennett D, Brown H, Bambra C, Taylor-Robinson D, Barr B (2021). Local government funding and life expectancy in England: a longitudinal ecological study. Lancet Public Health.

[86] Egan M, Kearns A, Mason P, et al. Protocol for a mixed methods study investigating the impact of investment in housing, regeneration and neighbourhood renewal on the health and wellbeing of residents: the GoWell programme. BMC Med Res Methodol. 2010;10(1). 10.1186/1471-2288-10-41. 10.1186/1471-2288-10-41 PMC2876178 20459767

[87] Arcaya MC, Graif C, Waters MC, Subramanian SV. Health selection into neighborhoods among families in the moving to opportunity program. Am J Epidemiol. 2016:kwv189. 10.1093/aje/kwv189. 10.1093/aje/kwv189 PMC4706680 26656481

[88] Fusar-Poli P, Radua J (2018). Ten simple rules for conducting umbrella reviews. Evid Based Ment Health.

