# Supplementary material for: Examining the effectiveness of place-based interventions to improve public health and reduce health inequalities: an umbrella review
Source: BMC Public Health. 2021 Oct 19;21:1888. doi: 10.1186/s12889-021-11852-z (PMC8524206; doi:10.1186/s12889-021-11852-z)
Supplement: Supplementary file 1 — Additional file 1. [file 12889_2021_11852_MOESM1_ESM.docx]

**Appendices**

***Appendix 1: PRISMA Checklist***

| **Section/topic** | **#** | **Checklist item** | **Reported on page #** |
| --- | --- | --- | --- |
| **TITLE** | | |  |
| Title | 1 | Identify the report as a systematic review, meta-analysis, or both. | 1 |
| **ABSTRACT** | | |  |
| Structured summary | 2 | Provide a structured summary including, as applicable: background; objectives; data sources; study eligibility criteria, participants, and interventions; study appraisal and synthesis methods; results; limitations; conclusions and implications of key findings; systematic review registration number. | 2 |
| **INTRODUCTION** | | |  |
| Rationale | 3 | Describe the rationale for the review in the context of what is already known. | 3-7 |
| Objectives | 4 | Provide an explicit statement of questions being addressed with reference to participants, interventions, comparisons, outcomes, and study design (PICOS). | 7-12 |
| **METHODS** | | |  |
| Protocol and registration | 5 | Indicate if a review protocol exists, if and where it can be accessed (e.g., Web address), and, if available, provide registration information including registration number. | 7 |
| Eligibility criteria | 6 | Specify study characteristics (e.g., PICOS, length of follow-up) and report characteristics (e.g., years considered, language, publication status) used as criteria for eligibility, giving rationale. | 9-12 |
| Information sources | 7 | Describe all information sources (e.g., databases with dates of coverage, contact with study authors to identify additional studies) in the search and date last searched. | 8-9 |
| Search | 8 | Present full electronic search strategy for at least one database, including any limits used, such that it could be repeated. | Appendix 2 |
| Study selection | 9 | State the process for selecting studies (i.e., screening, eligibility, included in systematic review, and, if applicable, included in the meta-analysis). | 12, Figure 1 |
| Data collection process | 10 | Describe method of data extraction from reports (e.g., piloted forms, independently, in duplicate) and any processes for obtaining and confirming data from investigators. | 12-13 |
| Data items | 11 | List and define all variables for which data were sought (e.g., PICOS, funding sources) and any assumptions and simplifications made. | 12-13 |
| Risk of bias in individual studies | 12 | Describe methods used for assessing risk of bias of individual studies (including specification of whether this was done at the study or outcome level), and how this information is to be used in any data synthesis. | 13 |
| Summary measures | 13 | State the principal summary measures (e.g., risk ratio, difference in means). | 13-14 |
| Synthesis of results | 14 | Describe the methods of handling data and combining results of studies, if done, including measures of consistency (e.g., I^2^) for each meta-analysis. | N/A |

Page 1 of 2

| **Section/topic** | **#** | **Checklist item** | **Reported on page #** |
| --- | --- | --- | --- |
| Risk of bias across studies | 15 | Specify any assessment of risk of bias that may affect the cumulative evidence (e.g., publication bias, selective reporting within studies). | 13 |
| Additional analyses | 16 | Describe methods of additional analyses (e.g., sensitivity or subgroup analyses, meta-regression), if done, indicating which were pre-specified. | N/A |
| **RESULTS** | | |  |
| Study selection | 17 | Give numbers of studies screened, assessed for eligibility, and included in the review, with reasons for exclusions at each stage, ideally with a flow diagram. | 14-17 Appendix 7 |
| Study characteristics | 18 | For each study, present characteristics for which data were extracted (e.g., study size, PICOS, follow-up period) and provide the citations. | 19-20 Appendix 5 |
| Risk of bias within studies | 19 | Present data on risk of bias of each study and, if available, any outcome level assessment (see item 12). | 18  Appendix 4 |
| Results of individual studies | 20 | For all outcomes considered (benefits or harms), present, for each study: (a) simple summary data for each intervention group (b) effect estimates and confidence intervals, ideally with a forest plot. | 19-20  Appendix 5 |
| Synthesis of results | 21 | Present results of each meta-analysis done, including confidence intervals and measures of consistency. | N/A |
| Risk of bias across studies | 22 | Present results of any assessment of risk of bias across studies (see Item 15). | N/A |
| Additional analysis | 23 | Give results of additional analyses, if done (e.g., sensitivity or subgroup analyses, meta-regression [see Item 16]). | N/A |
| **DISCUSSION** | | |  |
| Summary of evidence | 24 | Summarize the main findings including the strength of evidence for each main outcome; consider their relevance to key groups (e.g., healthcare providers, users, and policy makers). | 21-37 |
| Limitations | 25 | Discuss limitations at study and outcome level (e.g., risk of bias), and at review-level (e.g., incomplete retrieval of identified research, reporting bias). | 36-37 |
| Conclusions | 26 | Provide a general interpretation of the results in the context of other evidence, and implications for future research. | 37 |
| **FUNDING** | | |  |
| Funding | 27 | Describe sources of funding for the systematic review and other support (e.g., supply of data); role of funders for the systematic review. | 38 |

***Appendix 2: MEDLINE Search Strategy***

Database(s): **Ovid MEDLINE(R) and In-Process & Other Non-Indexed Citations** 1946 to February 28, 2020
Search Strategy:

| **#** | **Searches** | **Results** |
| --- | --- | --- |
| 1 | Social Planning/ or City Planning/ or Environment Design/ or Built Environment/ or Urban Renewal/ | 11878 |
| 2 | ((city or cities or town? or urban or rural$ or geographic$ or neighbourhood? or neighborhood? or local or regional$) adj1 (plan$ or renew$ or design$ or develop$ or regenerat$)).ti,ab,kw. | 19325 |
| 3 | ((area or place or location) adj (based or focused or centred or centered)).ti,ab,kw. | 3731 |
| 4 | ((local$ or region$) adj (approach$ or perspective?)).ti,ab,kw. | 1465 |
| 5 | or/1-4 | 34203 |
| 6 | Environmental Pollution/ae, lj, pc, st or exp Air Pollution/ae, lj, pc, st or exp Water Pollution/ae, lj, pc, st or Sanitation/ae, lj, pc, st | 40859 |
| 7 | Environmental Pollutants/ae, lj, pc, st or exp Air Pollutants/ae, lj, pc, st or exp Water Pollutants/ae, lj, pc, st | 22606 |
| 8 | exp Environmental Exposure/ae, lj, pc, st | 63315 |
| 9 | ((environment$ or air or water$) adj1 (pollut$ or quality or contamin$ or expose? or exposure? or health?)).ti,ab,kw. | 117277 |
| 10 | or/6-9 | 209536 |
| 11 | ((housing or residence? or workplace? or labor market? or labour market? or recreation$ or leisure or travel$ or transport$ or environment$) adj5 (health$ or unhealthy or safe$ or unsafe or secure or promot$)).mp. | 100531 |
| 12 | exp Socioeconomic Factors/ | 441058 |
| 13 | "Social Determinants of Health"/ | 2714 |
| 14 | Health Status Disparities/ | 14779 |
| 15 | Health Services Accessibility/ or Health Equity/ | 73170 |
| 16 | Healthcare Disparities/ | 15912 |
| 17 | Population Health/ | 725 |
| 18 | Health Behavior/ | 48796 |
| 19 | Health Promotion/ | 72216 |
| 20 | (wellbeing or (health adj1 (benefit$ or behavior$ or behaviour$ or promot$ or inequit$ or equit$ or inequalit$ or equalit$ or disparit$))).ti,ab,kw. | 115298 |
| 21 | (access$ adj2 (health$ or welfare or service?)).ti,ab,kw. | 27702 |
| 22 | or/12-21 | 685996 |
| 23 | (intervention$ or policy or policies or program$ or strateg$ or initiative$ or evaluat$ or effect$).ti,ab,kw. | 10152273 |
| 24 | 5 and 22 and 23 | 5717 |
| 25 | 10 and 22 and 23 | 7779 |
| 26 | 11 and 22 and 23 | 13831 |
| 27 | or/24-26 | 24067 |
| 28 | Developing Countries.sh,kf. | 84982 |
| 29 | (Africa or Asia or Caribbean or West Indies or South America or Latin America or Central America).hw,kf,ti,ab,cp. | 267651 |
| 30 | (Afghanistan or Albania or Algeria or Angola or Antigua or Barbuda or Argentina or Armenia or Armenian or Aruba or Azerbaijan or Bahrain or Bangladesh or Barbados or Benin or Byelarus or Byelorussian or Belarus or Belorussian or Belorussia or Belize or Bhutan or Bolivia or Bosnia or Herzegovina or Hercegovina or Botswana or Brasil or Brazil or Bulgaria or Burkina Faso or Burkina Fasso or Upper Volta or Burundi or Urundi or Cambodia or Khmer Republic or Kampuchea or Cameroon or Cameroons or Cameron or Camerons or Cape Verde or Central African Republic or Chad or Chile or China or Colombia or Comoros or Comoro Islands or Comores or Mayotte or Congo or Zaire or Costa Rica or Cote d'Ivoire or Ivory Coast or Croatia or Cuba or Cyprus or Czechoslovakia or Czech Republic or Slovakia or Slovak Republic or Djibouti or French Somaliland or Dominica or Dominican Republic or East Timor or East Timur or Timor Leste or Ecuador or Egypt or United Arab Republic or El Salvador or Eritrea or Estonia or Ethiopia or Fiji or Gabon or Gabonese Republic or Gambia or Gaza or Georgia Republic or Georgian Republic or Ghana or Gold Coast or Greece or Grenada or Guatemala or Guinea or Guam or Guiana or Guyana or Haiti or Honduras or Hungary or India or Maldives or Indonesia or Iran or Iraq or Isle of Man or Jamaica or Jordan or Kazakhstan or Kazakh or Kenya or Kiribati or Korea or Kosovo or Kyrgyzstan or Kirghizia or Kyrgyz Republic or Kirghiz or Kirgizstan or Lao PDR or Laos or Latvia or Lebanon or Lesotho or Basutoland or Liberia or Libya or Lithuania or Macedonia or Madagascar or Malagasy Republic or Malaysia or Malaya or Malay or Sabah or Sarawak or Malawi or Nyasaland or Mali or Malta or Marshall Islands or Mauritania or Mauritius or Agalega Islands or Mexico or Micronesia or Middle East or Moldova or Moldovia or Moldovian or Mongolia or Montenegro or Morocco or Ifni or Mozambique or Myanmar or Myanma or Burma or Namibia or Nepal or Netherlands Antilles or New Caledonia or Nicaragua or Niger or Nigeria or Northern Mariana Islands or Oman or Muscat or Pakistan or Palau or Palestine or Panama or Paraguay or Peru or Philippines or Philipines or Phillipines or Phillippines or Poland or Portugal or Puerto Rico or Romania or Rumania or Roumania or Russia or Russian or Rwanda or Ruanda or Saint Kitts or St Kitts or Nevis or Saint Lucia or St Lucia or Saint Vincent or St Vincent or Grenadines or Samoa or Samoan Islands or Navigator Island or Navigator Islands or Sao Tome or Saudi Arabia or Senegal or Serbia or Montenegro or Seychelles or Sierra Leone or Slovenia or Sri Lanka or Ceylon or Solomon Islands or Somalia or South Africa or Sudan or Suriname or Surinam or Swaziland or Syria or Tajikistan or Tadzhikistan or Tadjikistan or Tadzhik or Tanzania or Thailand or Togo or Togolese Republic or Tonga or Trinidad or Tobago or Tunisia or Turkey or Turkmenistan or Turkmen or Uganda or Ukraine or Uruguay or USSR or Soviet Union or Union of Soviet Socialist Republics or Uzbekistan or Uzbek or Vanuatu or New Hebrides or Venezuela or Vietnam or Viet Nam or West Bank or Yemen or Yugoslavia or Zambia or Zimbabwe or Rhodesia).hw,kf,ti,ab,cp. | 3594666 |
| 31 | ((developing or less* developed or under developed or underdeveloped or middle income or low* income or underserved or under served or deprived or poor*) adj (countr* or nation? or population? or world)).ti,ab. | 94628 |
| 32 | ((developing or less* developed or under developed or underdeveloped or middle income or low* income) adj (economy or economies)).ti,ab. | 508 |
| 33 | (low* adj (gdp or gnp or gross domestic or gross national)).ti,ab. | 236 |
| 34 | (low adj3 middle adj3 countr*).ti,ab. | 14506 |
| 35 | (lmic or lmics or third world or lami countr*).ti,ab. | 6843 |
| 36 | transitional countr*.ti,ab. | 156 |
| 37 | or/28-36 | 3744738 |
| 38 | 27 not 37 | 15798 |
| 39 | limit 38 to (english language and humans and yr="2008 -Current") | 9447 |
| 40 | letter/ | 1055473 |
| 41 | Editorial/ | 514161 |
| 42 | News/ | 198823 |
| 43 | exp Historical Article/ | 393978 |
| 44 | Anecdotes as Topic/ | 4734 |
| 45 | Comment/ | 830660 |
| 46 | (letter or comment$).ti. | 143408 |
| 47 | or/40-46 | 2418187 |
| 48 | 39 not 47 | 9294 |

***Appendix 3: Public Consultation***

Members of the public were advised this study seeks to understand what factors improve health and reduce inequalities in the places they live. Through a brief consultation exercise people were asked to list factors they felt have a positive or negative impact on health, wellbeing, and inequalities in their area. The following table indicates their responses.

| **Role** | **Comments** |
| --- | --- |
| Public – residents and VCO representatives | Positive  Community allotment, sustainable food production – deliver to local foodbank and community café.  Increase coordination of VCOs providing youth services and social enterprise support, community events, and environmental improvements.  VCOs organised community litter picks, community events and festivals, social media sharing good news stories.  Neighbours are helpful, volunteering opportunities, VCOs organised activities – community spirit.  VCOs mindful walks, drop in coffee mornings, cycle rides, green spaces.  VCOs activities: walking group, bike club, litter picking events, craft clubs, providing leisure passes for 3 months to local residents. Community allotment – used by care homes, schools and local residents.  Collaboration of local organisations thinking about how to improve services – eg. Youth provision but needs to go further.  The coast, the parks, park run, walks along the coast, clean streets.  Community buildings lots of activities – craft, crèche, keep fit, activities for all ages – VCOs organised.  Good public transport, VCO activities in community centre.  VCOs – small grants scheme to help community projects.  VCOs – community centre that targets ASB and engages multi agency approach to drugs and prevention. Litter picks. |
|  | Negative  Quad bike riding on open spaces/parks and fly tipping.  Lack of support and engagement from LA, decrease in health services and youth services in the area. Dog fouling – low level antisocial behaviour.  Increased antisocial behaviour by young people (real and perceived).  Fly-tipping. Social media – gossiping and negativity.  Car parking, lack of facilities for disabled people – swings in parks. Off-road motorbikes/quads. Overgrown footpaths, litter, parking outside schools, roofing on houses need renewing – causing problems. Dogs barking. Graffiti.  Fly tipping, antisocial behaviour.  Lots of housing being lost. Community centres and libraries being lost.  Roll out of universal credit is affecting not just unemployed but creating working poor, less resources and more unhealthy.  Dog fouling.  Traffic in town centre, poor temp accommodation.  Fly-tipping – in streets and parks, dog fouling – no one around to implement fines. Sure start centres – all closed. Older children – nothing for them to do on weekends and evenings – hanging around streets, parks, or shops. Pot holes in most streets.  Lots of fly tipping – seems to be getting worse. Lots of dog fouling.  Lots of cars and motor bikes racing around the streets late at night. Lighting bad in places at night, don’t feel safe to be outside.  Youth clubs closed and need more for young people to do.  Increase in dog fouling, ABS – youngsters who are “too cool” to go to the youth club and are “bored”. |

***Appendix 4: Data extraction and Quality Appraisal template***

| **Bibliographical** **details** | | | | | | | | | | | | | | | | | | | |
| --- | --- | --- | --- | --- | --- | --- | --- | --- | --- | --- | --- | --- | --- | --- | --- | --- | --- | --- | --- |
| **Author** |  | | | | | | | | | | | | | | | | | | |
| **Year** |  | | | | | | | | | | | | | | | | | | |
| **Title** |  | | | | | | | | | | | | | | | | | | |
|  | | | | | | | | | | | | | | | | | | | |
| **Review details** | | | | | | | | | | | | | | | | | | | |
| **Systematic review based on DARE Criteria?**  1) a clear question;  2) a transparent method for the search, selection and appraisal of evidence or studies;  3) a synthesis of results or evidence | | | | | | | | | | | 1. Yes/no 2. Yes/no 3. Yes/no   Only continue if all 3 of the above criteria are met, if the review does not meet these criteria it will be excluded (add details for exclusion below) | | | | | | | | |
| **Population**  (any age/gender/location etc) | | | | | | | | | | |  | | | | | | | | |
| **Intervention(s)**  Clear, succinct details of the interventions of interest should be described, including the type of intervention, the frequency and/or intensity of the intervention for example. | | | | | | | | | | |  | | | | | | | | |
| **Place-based setting/context**  Physical, social, or economic environment and country/region | | | | | | | | | | |  | | | | | | | | |
| **Health/health inequalities outcome(s)** | | | | | | | | | | |  | | | | | | | | |
| **Cost-effectiveness data** | | | | | | | | | | |  | | | | | | | | |
| **Variations in outcome(s) between population groups (using PROGRESS+ Factors)** | | | | | | | | | | |  | | | | | | | | |
| **Number of databases/sources searched** | | | | | | | | | | |  | | | | | | | | |
| **Number of relevant studies in review (total)**  Only studies reporting post-2008 interventions should be included in this total. | | | | | | | | | | |  | | | | | | | | |
| **Date range of included studies**  The date range spanning from the earliest study that informs the included research synthesis to the latest should be reported. If this is not readily identifiable in the table of study characteristics provided by the included synthesis, it should be discernible by scanning the date range of publications through the results section of the included review. **Can report full date range but only extract from post-2008 interventions.** | | | | | | | | | | |  | | | | | | | | |
| **Study design of included studies**  e.g. RCTS, Non-randomised controlled trials, controlled prospective cohort, repeat cross sections, natural experiments, observational studies etc. | | | | | | | | | | |  | | | | | | | | |
| **Method of synthesis**  The type of research synthesis as stated by the authors of the included review should be detailed e.g. random effects meta-analysis, fixed effect meta-analysis, meta- aggregative synthesis, meta-ethnography, or narrative synthesis. | | | | | | | | | | |  | | | | | | | | |
| **Quality** (as measured by systematic review authors)  The instrument or tool used to assess risk of bias, rigor or study quality should be reported along with some summary estimate of the quality of primary studies in the included research synthesis. | | | | | | | | | | |  | | | | | | | | |
| **Are all included studies relevant for this umbrella review?** | | | | | **Y/N** | | | | | | If Y, go to ‘Summary table’; if N, complete ‘Primary studies findings’ | | | | | | | | |
| **Primary studies findings** | | | | | | | | | | | | | | | | | | | |
| For relevant studies in the review summaries: | | | | | | | | | | | | | | | | | | | |
| **Study No.** | **Author(s) of original publication**^[[1]](#footnote-1)^  **Intervention**  **Relevant page numbers** | | | | **Study design** | | **Setting and participants**^[[2]](#footnote-2)^ | | **Intervention description** | | **Health/health inequalities outcome(s)** | | **PROGRESS +** | **Summary results**  **Description and summary results**  **↑ = increase/ improved**  **↓ = decrease/ deteriorated**  **↔ = no change** | | **Quality appraisal**^[[3]](#footnote-3)^ | | **Impact on health/health inequalities** | |
|  |  | | | |  | |  | |  | |  | |  |  | |  | |  | |
|  |  | | | |  | |  | |  | |  | |  |  | |  | |  | |
|  |  | | | |  | |  | |  | |  | |  |  | |  | |  | |
|  |  | | | |  | |  | |  | |  | |  |  | |  | |  | |
|  |  | | | |  | |  | |  | |  | |  |  | |  | |  | |
| **Summary table** | | | | | | | | | | | | | | | | | | | |
| **Study** | | **No. of relevant studies** | **Context (setting, country, search timeframe)** | | | | | | | **Intervention(s)** *If more than 1 intervention, enter n of studies* | | | | | **Summary of results** | | | | **R-AMSTAR quality appraisal** |
|  | |  |  | | | | | | |  | | | | |  | | | |  |
|  | |  |  | | | | | | |  | | | | |  | | | |  |
|  | |  |  | | | | | | |  | | | | |  | | | |  |
|  | | | | | | | | | | | | | | | | | | | |
| **Summary paragraph** | | | | | | | | | | | | | | | | | | | |
|  | | | | | | | | | | | | | | | | | | | |
|  | | | | | | | | | | | | | | | | | | | |
| **AMSTAR tool for assessing methodological quality of systematic reviews** | | | | | | | | | | | | | | | | | | | |
|  | | | | | | | | **YES/NO/Can’t answer/N/A** | | | | **R-AMSTAR checklist** | | | | | **R-AMSTAR points** | | |
| **1. Was an ‘‘a priori’’ design provided?**  The research question and inclusion criteria should be established before the conduct of the review.  *Note: need to refer to a protocol, ethics approval, or pre-determined/a priori published research objectives to score a “yes”* | | | | | | | |  | | | | A: ‘a priori design’  B: statement of inclusion criteria  C: PICO research question  If it satisfies 3 of the criteria → 4  If it satisfies 2 of the criteria → 3  If it satisfies 1 of the criteria → 2  If it satisfies 0 of the criteria → 1 | | | | |  | | |
| **2. Was there duplicate study selection and data extraction?**  There should be at least two independent data extractors and a consensus procedure for disagreements should be in place.  Note: 2 people do study selection, 2 people do data extraction, consensus process or one person checks the other’s work. | | | | | | | |  | | | | A: There should be at least two independent data extractors as stated or implied.  B: Statement of recognition or awareness of consensus procedure for disagreements.  C: Disagreements among extractors resolved properly as stated or implied.  If it satisfies 3 of the criteria →4  If it satisfies 2 of the criteria →3  If it satisfies 1 of the criteria →2  If it satisfies 0 of the criteria →1 | | | | |  | | |
| **3. Was a comprehensive literature search performed?**  At least two electronic sources should be searched. The report must include years and databases used (e.g., Central, EMBASE, and MEDLINE). Key words and/or MESH terms must be stated and where feasible the search strategy should be provided. All searches should be supplemented by consulting current contents, reviews, textbooks, specialized registers, or experts in the particular field of study, and by reviewing the references in the studies found.  Note: If at least 2 sources + one supplementary strategy used, select “yes” (Cochrane register/Central counts as 2 sources; a grey literature search counts as supplementary). | | | | | | | |  | | | | A: At least two electronic sources should be searched.  B: The report must include years and databases used (e.g. Central, EMBASE, and MEDLINE).  C: Key words and/or MESH terms must be stated AND where feasible the search strategy outline  should be provided such that one can trace the filtering process of the included articles.  D: In addition to the electronic databases (PubMed, EMBASE, Medline), all searches should be supplemented by consulting current contents, reviews, textbooks, specialized registers, or experts  in the particular field of study, and by reviewing the references in the studies found.  E: Journals were “hand‐searched” or “manual searched” (i.e. identifying highly relevant journals  and conducting a manual, page‐by‐page search of their entire contents looking for potentially  eligible studies).  If it satisfies 4 or 5 of the criteria → 4  If it satisfies 3 of the criteria → 3  If it satisfies 2 of the criteria →2  If it satisfies 1 or 0 of the criteria → 1 | | | | |  | | |
| **4. Was the status of publication (i.e. grey literature) used as an inclusion criterion?**  The authors should state that they searched for reports regardless of their publication type. The authors should state whether or not they excluded any reports (from the systematic review), based on their publication status, language etc.  Note: If review indicates that there was a search for “grey literature” or “unpublished literature,” indicate  “yes.” SINGLE database, dissertations, conference proceedings, and trial registries are all considered grey for this purpose. If searching a source that contains both grey and non‐grey, must specify that they were searching for grey/unpublished lit. | | | | | | | |  | | | | A: The authors should state that they searched for reports regardless of their publication type.  B: The authors should state whether or not they excluded any reports (from the systematic  review), based on their publication status, language etc.  C: “Non‐English papers were translated” or readers sufficiently trained in foreign language.  D: No language restriction or recognition of non‐English articles.  If it satisfies 3 or 4 of the criteria →4  If it satisfies 2 of the criteria →3  If it satisfies 1 of the criteria →2  If it satisfies 0 of the criteria →1 | | | | |  | | |
| **5. Was a list of studies (included and excluded) provided?**  A list of included and excluded studies should be provided.  Note: Acceptable if the excluded studies are referenced. If there is an electronic link to the list but the link is dead, select “no.” | | | | | | | |  | | | | A: Table/list/or figure of included studies, a reference list does not suffice.  B: Table/list/figure of excluded studies1 either in the article or in a supplemental source (i.e.  online). (Excluded studies refers to those studies seriously considered on the basis of title and/or  abstract, but rejected after reading the body of the text).  C: Author satisfactorily/sufficiently stated the reason for exclusion of the seriously considered  studies.  D: Reader is able to retrace the included and the excluded studies anywhere in the article  bibliography, reference, or supplemental source.  If it satisfies 4 of the criteria →4  If it satisfies 3 of the criteria →3  If it satisfies 2 of the criteria →2  If it satisfies 1 or 0 of the criteria → 1 | | | | |  | | |
| **6. Were the characteristics of the included studies provided?**  In an aggregated form such as a table, data from the original studies should be provided on the participants, interventions and outcomes. The ranges of characteristics in all the studies analyzed e.g., age, race, sex, relevant socioeconomic data, disease status, duration, severity, or other diseases should be reported.  Note: Acceptable if not in table format as long as they are described as above. | | | | | | | |  | | | | A: In an aggregated form such as a table, data from the original studies should be provided on the  participants, interventions AND outcomes.  B: Provide the ranges of relevant characteristics in the studies analyzed (e.g. age, race, sex,  relevant socioeconomic data, disease status, duration, severity, or other diseases should be  reported.)  C: The information provided appears to be complete and accurate (i.e. there is a tolerable range of subjectivity here. Is the reader left wondering? If so, state the needed information and the reasoning).  If it satisfies 3 of the criteria →4  If it satisfies 2 of the criteria →3  If it satisfies 1 of the criteria →2  If it satisfies 0 criteria → 1 | | | | |  | | |
| **7. Was the scientific quality of the included studies assessed and documented?**  'A priori' methods of assessment should be provided (e.g., for effectiveness studies if the author(s) chose to  include only randomized, double‐blind, placebo controlled studies, or allocation concealment as inclusion criteria); for other types of studies alternative items will be relevant.  Note: Can include use of a quality scoring tool or checklist, e.g., Jadad scale, risk of bias, sensitivity analysis, etc., or a description of quality items, with some kind of result for EACH study (“low” or “high” is fine, as long as it is clear which studies scored “low” and which scored “high”; a summary score/range for all studies is not acceptable). | | | | | | | |  | | | | A: ‘A priori’ methods of assessment should be provided (e.g., for effectiveness studies if the  author(s) chose to include only randomized, double‐blind, placebo controlled studies, or allocation concealment as inclusion criteria); for other types of studies alternative items will be relevant.  B: The scientific quality of the included studies appears to be meaningful.  C: Discussion/recognition/awareness of level of evidence.  D: Quality of evidence should be rated/ranked based on characterized instruments. (Characterized instrument is a created instrument that ranks the level of evidence, e.g. GRADE [Grading of Recommendations Assessment, Development and Evaluation.])  If it satisfies 4 of the criteria →4  If it satisfies 3 of the criteria →3  If it satisfies 2 of the criteria →2  If it satisfies 1 or 0 of the criteria →1 | | | | |  | | |
| **8. Was the scientific quality of the included studies used appropriately in formulating conclusions?**  The results of the methodological rigor and scientific quality should be considered in the analysis and the  conclusions of the review, and explicitly stated in formulating recommendations.  Note: Might say something such as “the results should be interpreted with caution due to poor quality of  included studies.” Cannot score “yes” for this question if scored “no” for question 7. | | | | | | | |  | | | | A: The results of the methodological rigor and scientific quality should be considered in the  analysis and the conclusions of the review  B: The results of the methodological rigor and scientific quality are explicitly stated in formulating  recommendations.  C: To have conclusions integrated/drives towards a clinical consensus statement.  D: This clinical consensus statement drives toward revision or confirmation of clinical practice  Guidelines.  If it satisfies 4 of the criteria →4  If it satisfies 3 of the criteria →3  If it satisfies 2 of the criteria →2  If it satisfies 1 or 0 of the criteria → 1 | | | | |  | | |
| **9. Were the methods used to combine the findings of studies appropriate?**  For the pooled results, a test should be done to ensure the studies were combinable, to assess their  homogeneity (i.e., Chi‐squared test for homogeneity, I2). If heterogeneity exists a random effects model  should be used and/or the clinical appropriateness of combining should be taken into consideration (i.e., is it sensible to combine?).  Note: Indicate “yes” if they mention or describe heterogeneity, i.e., if they explain that they cannot pool because of heterogeneity/variability between interventions. | | | | | | | |  | | | | A: Statement of criteria that were used to decide that the studies analyzed were similar enough to  be pooled?  B: For the pooled results, a test should be done to ensure the studies were combinable, to assess  their homogeneity (i.e. Chi‐squared test for homogeneity, I^2^).  C: Is there a recognition of heterogeneity or lack of thereof.  D: If heterogeneity exists a “random effects model” should be used and/or the rationale (i.e.  clinical appropriateness) of combining should be taken into consideration (i.e. is it sensible to  combine?), or stated explicitly.  E: If homogeneity exists, author should state a rationale or a statistical test.  If it satisfy 4 or 5 of the criteria → 4  If it satisfy 3 of the criteria → 3  If it satisfy 2 of the criteria →2  If it satisfy 1 or 0 of the following criteria → 1 | | | | |  | | |
| **10. Was the likelihood of publication bias assessed?**  An assessment of publication bias should include a combination of graphical aids (e.g., funnel plot, other  available tests) and/or statistical tests (e.g., Egger regression test, Hedges‐Olken).  Note: If no test values or funnel plot included, score “no”. Score “yes” if mentions that publication bias could not be assessed because there were fewer than 10 included studies. | | | | | | | |  | | | | A Recognition of publication bias or file‐drawer effect.  B: An assessment of publication bias should include graphical aids (e.g., funnel plot, other  available tests).  C: Statistical tests (e.g. Egger regression test).  If it satisfies 3 of the criteria →4  If it satisfies 2 of the criteria →3  If it satisfies 1 of the criteria →2  If it satisfies 0 of the criteria →1 | | | | |  | | |
| **11. Was the conflict of interest included?**  Potential sources of support should be clearly acknowledged in both the systematic review and the included studies.  Note: To get a “yes,” must indicate source of funding or support for the systematic review AND for each of the included studies. | | | | | | | |  | | | | A: Statement of sources of support.  B: No conflict of interest. This is subjective and may require some deduction or searching.  C: An awareness/statement of support or conflict of interest in the primary inclusion studies.  If it satisfies 3 of the criteria →4  If it satisfies 2 of the criteria →3  If it satisfies 1 of the criteria →2  If it satisfies 0 of the criteria →1 | | | | |  | | |
|  | | | | | | | | **TOTAL:** | | | | Maximum 44 | | | | |  | | |
|  | | | | | | | |  | | | | Quality appraisal:  Low = 11 ‐22  Medium = 23‐33  High = 34 ‐ 44 | | | | |  | | |
| **Data extractor record** | | | | | | | | | | | | | | | | | | | |
| **Name of data extractor** | | | | **Date** | | **Comments** | | | | | | | | | | | | | |
|  | | | |  | |  | | | | | | | | | | | | | |

***Appendix 5: Study summary table***

| **Study** | **No. of relevant studies (no. of studies included in original review).** | **Context (country, population, search timeframe)** | **Intervention(s)** | **Outcomes of relevant studies.** | **Summary of results (includes quality assessment by original review authors).** | **R-AMSTAR quality appraisal rating** |
| --- | --- | --- | --- | --- | --- | --- |
| Brown et al (2015)^54^ | 1 (12) | London, Singapore, Mila, Stockholm, or Gothenburg; no restrictions on population; inception to 2015 | **Economic:**  Traffic congestion pricing scheme. Cordon pricing, time-differentiated charges, weekday travel. | Health behaviours; active transport.  No variation reported in PROGRESS+ factors. | **Traffic congestion pricing scheme.**  Moderate quality evidence suggests traffic congestion pricing schemes leads to increases in health-related behaviours such as shifting from car trips to public transport. Börjesson and Kristoffersson (2015) reported a 24% increase in the total number of trips by public transport, a 9% decrease in commuter car trips, a decrease in all discretionary trips, and a 36% decrease in commuter cycling trips after traffic congestion charges were introduced in Gothenburg. Authors noted these findings were unreliable due to adverse weather conditions, and a small and unrepresentative sample. | 26 (medium) |
| Audrey & Batista-Ferrer (2015)^16^ | 2 (33) | any high-income country, urban environments; children and young people; inception to 2014. | **Physical:**  Park intervention – manipulation of seating location around park.  Smart Growth Community: assesses whether children change where they engage in physical activity after moving to Smart Growth community. | Health behaviours; physical activity.  No reported variation in PROGRESS+ factors. | **Manipulation of seating location in park playground.**  Low quality evidence suggests removing seating arrangements in parks did not change the likelihood of children standing or engaging in moderate to vigorous physical activity.  **Relocation to Smart Growth community.**  Smart Growth community -greater building density, less auto-dominated form, greater non-residential land uses, fewer barriers to connectivity, more parks and playgrounds, more traffic safety and aesthetic features, and fewer physical incivilities such as graffiti and litter – compared to control.  Study reported no strong evidence for increase in moderate to vigorous physical activity in the Smart Growth group  than the control group. Moderate quality. | 31 (medium) |
| Hunter et al (2015)^55^ | 3 (12) | No country restrictions; any population; inception to 2014 | **Physical:**  Development of new greenway.  Addition of family fitness zones (outdoor gyms).  Park re-development. | Health behaviours; physical activity.  No reported variation in PROGRESS+ factors. | **Development of new greenway.**  High risk of bias. No significant impact on physical activity between intervention and control group. In both groups there were increases in mean number of days respondents walked or were moderately active.  **Family fitness zones (outdoor gyms).**  Unclear risk of bias. Non-significant increases in park usage by 11% reported in intervention parks compared to control. Self-reports of being a new park user increased more in intervention parks in addition to higher estimated energy expenditure at both follow-ups than at baseline. 29% of households in poverty, 59% Latino population – no assessment of variation based on these characteristics.  **Park re-development.**  Unclear risk of bias. Significant increase in number of park users from pre- to post-improvement and increases in number of people walking and being vigorously active. Implemented in most disadvantaged decile in state of Victoria, Australia. | 22 (low) |
| Mayne et al (2015)^56^ | 4 (37)  (3 studies extracted from Hunter et al, 2015) | No country restrictions; any population; 2005-2014. | **Physical: New parks, active transport; bike lanes and bike share schemes.** | Health behaviours; physical activity.  No reported variation in PROGRESS+ factors. | **New park playground and improvements.**  Intermediate study design (++). Direction of evidence reported as null. No difference in observed differences in physical activity between the intervention and control park.  **Active transport: Bicycle share scheme.**  Intermediate study design (++). Direction of evidence reported as expected. Greater odds of cycling among those exposed to the bike share program than a comparison group.  **Active transport: Bike lanes.**  Low (+) and intermediate study design (++). Direction of evidence expected. Increase in average number of cyclists per day after bike lanes implemented and larger increases in cyclists in streets with new bike lanes. | 21 (low) |
| Sauni et al (2015)^57^ | 1 (12) | No country restrictions; adults and children in buildings damaged by water or moulds; inception to 2014. | **Physical:**  **Home modifications** | Health outcomes; Asthma.  No reported variation in PROGRESS+ factors. | **Home modifications.**  Poor quality evidence showed asthma-related outcomes did not decrease after homes were repaired after a flood compared to those living in homes that had already been repaired. | 41 (high) |
| McCartney et al (2017)^17^ | 2 (46) | High income countries; no restrictions on population reported; inception to 2016. | **Physical, social, and economic:**  **Regeneration programme and housing modification (physical only).** | Health outcomes: self-reported physical and mental health; perception of area. No reported variation in PROGRESS+ factors. | **Housing modification.**  Respondents reported improved self-rated health and mental health after housing refurbishments focussed on energy efficient boilers and double glazing. Use of health care services also reduced post-intervention. Review authors classified this evidence as low quality as there was no comparison group, and no response/attrition rates were reported.  **Housing, physical environment, employment training, and community spaces.**  Respondents reported improved perception of the area after an urban renewal programme, that involved housing refurbishment, improvements to the physical environment, external maintenance, community engagement, employment training, and creation of community spaces. There was no change in self-reported health. Review authors classified this evidence as low quality as no comparison group and attrition was not reported, however, there were high response rates. | 17 (low) |
| Macmillan et al (2018)^58^ | 6 (15)  (3 studies extracted from Hunter et al, 2015 and Foster et al, 2017) | No country restrictions; any population; inception to 2017. | **Physical: Cycle lanes, supermarkets, transport.** | Health behaviours; physical activity, consumption of fruit and vegetables.  Health outcomes; self-reported BMI.  No reported variation in PROGRESS+ factors. | **Installation of new bike boulevards.**  Bicycle boulevard introduction was negatively correlated with bicycling and the number of bike trips (risk of bias 5/9).  **New supermarket/farmers market.**  No significant changes in self-report BMI were reported after the introduced of a new supermarket (risk of bias 5/9).  The introduction of community farmers market 1 day a week was associated with self-reported increase in purchases and consumption of fresh fruit and vegetables (risk of bias 2/9).  **New light rail transit.**  Negative association between total walk trips based on the interaction of distance to rail stop and baseline walking trips. Increase in physical activity for new users of light rail transit (risk of bias 4/9).  **New light rail transit and bike/walking routes.**  Decrease in physical activity for former riders of light rail transit. Increase in sedentary behaviour for former riders, greater than those never using light rail transit. Increased physical activity greater for those living in closer proximity to the intervention (risk of bias 6/9). | 34 (high) |
| Moore et al (2018)^59^ | 3 (14) | Urban environments in high income countries; Adults and older adults; inception to 2016. | **Physical: Guided busway, green storm water infrastructure, urban and landscape development.** | Health behaviours; physical activity.  Health outcomes; quality of life, perceptions of safety and social cohesion.  No reported variation in PROGRESS+ factors. | **Guided busway.**  Purpose-built guided segregated bus track with cycle and walking path. Mental health outcomes improved for respondents who used the new route for active commuting (cycling/walking). However, this effect attenuated when controlled from baseline mental health. Risk of bias not reported.  **Green storm water infrastructure.**  Landscape changes to improve watershed function and sewer stormwater capacity by planting trees, repair and replacement of sewer pipe and addition of stormwater facilities in public right of way areas. No effect of improvements to green infrastructure on whether people felt the neighbourhood was friendly or sociable and no effect on fear-of-crime when compared to control site. Moderate risk of bias.  **Urban and landscape development.**  Streets were redesigned to look more attractive and safer with buildouts to slow traffic, planters, benches, and light. People living in intervention streets improved quality of life by average 3.2 points compared with a fall by 7.25 points in control streets. This represents a medium effect size but the broad 95% confidence intervals include the null and with range of size of effect from very small increase to substantial decrease in quality of life. Serious risk of bias. | 36 (high) |
| Stappers et al (2018)^60^ | 7 (19) | No country restrictions; adults; inception to 2018. | **Physical:** New on- and off-road walking/cycling routes. | Health behaviours: Physical activity.  No reported variation in PROGRESS+ factors. | **Cycling and walking routes.**  Critical to moderate risk of bias.  West & Shore (2015) reported no sig changes in physical activity after an additional 1.93 miles of greenway was added to an existing greenway.  Crane et al (2017), Heesch et al (2016) and Rissel (2015) reported increase in cycling after construction of new separated bicycle paths.  The implementation of a traffic free bridge (Song et al, 2017) and a busway with parallel walking and cycling trail (Heinen et al, 2015; 2017) resulted in non-significant and negative effects on overall physical activity, walking, and cycling.  Crane et al (2017) reported living in close proximity to the intervention (between 1.0-2.99km from the intervention area was associated with a higher increase of cycling compared with individuals living closer (<1.0km) or further away (>3.0km). Heinen et al (2017) and Song et al (2017) found living closer to the busway and parallel walking/cycling route was associated with more walking and cycling. | 28 (medium) |
| Tseng et al (2018)^61^ | 2 (17) | No country restrictions; adults; 2000-2017 | **Physical:**  **Opening new supermarket** | Health behaviours; consumption of fruit and vegetables.  Health outcomes; weight/BMI  No reported variation in PROGRESS+ factors. | **New supermarket**  One low risk of bias study reported no difference in fruit and vegetable consumption after the construction of a new supermarket compared to a control neighbourhood. Decrease in overall caloric intake for intervention neighbourhood. Non-significant reduced in BMI for intervention group.  One high risk of bias study reported reduced BMI in intervention town after opening of new grocery store in centre of neighbourhood that lacked access to healthy food where no other store available within walking distance. Increase in BMI in control town. | 33 (medium) |
| Hunter et al (2019)^51^ | 6 (43) | No country restrictions; no population restrictions; 2002-2016 | **Physical: New, or renovation of existing, parks; development of vacant lots; outdoor gym.** | Health behaviours; physical activity.  Health outcomes; heart rate,  Social determinants outcomes; gun assaults, perceptions of safety, crime rates.  Lack of PROGRESS+ data available in primary studies to assess variation in outcomes. | **Development of new parks.**  Significant increase in total number of people observed using the park post-intervention (p=0.004); increase in proportion of users engaging in moderate (p=0.007) or vigorous PA (p=0.04).Post-intervention average monthly visitors sig increased (p=0.002). QA: 9/11  **Existing park renovations.**  One study examined impact of replacing old playground equipment and ground surfacing. Control parks with no renovations, parks were matched on size, proximity, neighbourhood SES, race and ethnicity. Significant increases between baseline and 12 month follow up for park utilization and the number of people engaged in MVPA; increase in park utilization over time in intervention parks compared with control. QA: 9/11  One study examined soft measures in park renovations which included signage (e.g. banners, walking path signs), promotional incentives (e.g. water bottles, park-branded key chains, individually targeted emails), and outreach activities (e.g. hiring community engagement officers, buying activity materials). Physical activity increased in intervention parks, generating an estimated average of 600 more visits/week/park, and 1830 more MET-hours of physical activity/week/park. Cost-effectiveness based on increased physical activity, measured in metabolic equivalent of task (MET)-hours/year. Each MET-hour gained is equivalent to a person engaging in moderate to vigorous PA for approx. 15min, with CE judged at whether the cost was less than between $0.50 and $1.00 per MET-hour. CE was reported to be $0.14 to $2.40 per MET. QA: 11/11  **Greening of vacant lots.**  One Study examined greening of vacant lots which included removing debris, grading the land and adding topsoil, planting grass and trees, building a wooden fence. Control – no greening intervention (4500-5500 square feet of vacant lots identified as intervention of control sites; 50 index sites in total). Non-significant decrease in the number of total crimes and gun assaults around greened vacant lots compared with control; people around the intervention lots reported feeling sig safer after the greening intervention compared with control lots (p<0.01). QA:11/11  One study examined the impact of greening vacant lots on heart rate among African Americans. Randomly selected cluster of vacant lots received standard greening treatment involving cleaning and removing debris, planting grass and trees, and installing a low wooden post-and-rail fence. Control – randomly selected cluster of vacant lots did not receive the greening treatment. Significant reduction in heart rate in African Americans exposed to greened compared to non-greened vacant lots. Difference in difference estimates between greened and non-greened vacant lots was sig lower for heart rate (p<0.001) for the greened site; being in view of a greened vacant lot decreased heart rate sig more than non-greened lot. QA 7/11  **Installation of outdoor gym.**  Cranney et al, 2016: Outdoor gym installed (60,000 Aus$), targeted marketing and promotional strategies to engage older adults and hosting exercise sessions by a professional. Park is 16.08 ha, with direct access to picnic shelters, barbecues, drinking foundations, toilets and change facilities, a skate park and children’s playground. Small but sig increase in senior park users engaging in MVPA at follow up (1.6 to 5.1%; p<0.001); sig increases from baseline to follow-up in the outdoor gym area for MVPA (6 to 40%; p,0.001); and seniors’ use (1.4 to 6%; p,0.001) QA: 7/11. | 36 (high) |
| Ige et al (2019)^52^ | 1 (16) | No country restrictions; any population; inception to 2017 | **Physical: Housing modifications.** | Health outcomes; injuries from falls.  No reported variation in PROGRESS+ factors. | **Housing modifications.**  High quality evidence shows a 26% reduction in the rate of home injuries caused by falls in the group that received home modifications. Injuries specific to the intervention also declined by 39% per year among the intervention group. | 23 (medium) |
| Persaud et al (2019)^53^ | 13 (59) | No country restrictions; any population; 1995 to 2017. | **Physical: Housing First interventions** | Health outcomes; 34 different outcomes.  No reported variation in PROGRESS+ factors, however, all studies included people with multiple disadvantage (homeless, substance misuse). | **Housing First interventions.**  Thirteen studies reported ‘Housing First’ interventions which combined the provision of housing with the addition of treatment for various addictions, mental health challenges and other social supports. 11 studies had intervention and treatment as usual groups. 2 studies had before and after housing intervention.  Study durations ranged from 6 to 180 months and reported a total of 34 outcomes of which 31 were statistically significant and 3 had unknown significance. Of the 31 statistically significant outcomes 12 outcomes (from 12 studies) favoured the intervention, and 19 outcomes (from 11 studies) favoured the control.  Although results were mixed with 19 outcomes favouring the control, all control groups had treatment for addiction, mental health challenges, and other social supports but limited detail was provided on these to contextualise the findings. Duration of studies varied and this may provide insight into the differing outcomes for intervention and control groups.  Results suggest provision of housing can promote health in certain circumstances. | 33 (medium) |

***Appendix 6: Reasons for exclusion of full-text articles***

***Not a systematic review (n=126)***

1. South J, Jackson K, Warwick-Booth L. The community health apprentices project - the outcomes of an intermediate labour market project in the community health sector. *Community, Work Fam*. 2011;14(1):1-18.

2. Monks R, Robertson S. Evaluation of a neighbourhood health and wellbeing programme: Rob Monks, Steve Robertson and Gulab Singh outline the findings of an evaluation of the health and wellbeing aspect of Neighbourhood Renewal Fund programmes. *Prim Heal Care*. 2009;19(3):34-38.

3. Wicks-Lim J, Arno PS. Improving population health by reducing poverty: New York’s Earned Income Tax Credit. *SSM - Popul Heal*. 2017;3:1-10.

4. Olsen JR, Mitchell R, Ogilvie D, team M study. Effects of new motorway infrastructure on active travel in the local population: a retrospective repeat cross-sectional study in Glasgow, Scotland. *Int J Behav Nutr Phys Act*. 2016;13:77.

5. Cheadle A, Atiedu A, Rauzon S, et al. A Community-Level Initiative to Prevent Obesity: Results From Kaiser Permanente’s Healthy Eating Active Living Zones Initiative in California. *Am J Prev Med*. 2018;54(5):S150-S159.

6. Arthurson K, Levin I, Ziersch A. Public housing renewal and social determinants of health. *J Prev Interv Community*. 2016;44(4):233-246.

7. Foley L, Prins R, Crawford F, et al. Effects of living near an urban motorway on the wellbeing of local residents in deprived areas: Natural experimental study. *PLoS ONE [Electronic Resour*. 2017;12(4):e0174882.

8. Torres A, Steward J, Strasser S, Lyn R, Serna R, Stauber C. Atlanta Streets Alive: A Movement Building a Culture of Health in an Urban Environment. *J Phys Act Health*. 2016;13(2):239-246.

9. Breysse J, Dixon SL, Jacobs DE, Lopez J, Weber W. Self-reported health outcomes associated with green-renovated public housing among primarily elderly residents. *J Public Heal Manag Pract*. 2015;21(4):355-367.

10. Lomas J, Schmitt L, Jones S, et al. A pharmacoeconomic approach to assessing the costs and benefits of air quality interventions that improve health: a case study. *BMJ Open*. 2016;6(6):e010686.

11. Grey CN, Jiang S, Nascimento C, et al. The short-term health and psychosocial impacts of domestic energy efficiency investments in low-income areas: a controlled before and after study. *BMC Public Health*. 2017;17(1):140.

12. Anderson J, Ruggeri K, Steemers K, Huppert F. Lively Social Space, Well-Being Activity, and Urban Design: Findings From a Low-Cost Community-Led Public Space Intervention. *Environ Behav*. 2017;49(6):685-716.

13. Parker KM, Rice J, Gustat J, Ruley J, Spriggs A, Johnson C. Effect of bike lane infrastructure improvements on ridership in one New Orleans neighborhood. *Ann Behav Med*. 2013;45:S101-7.

14. Hansji NL, Wilson NJ, Cordier R. Men’s Sheds: enabling environments for Australian men living with and without long-term disabilities. *Health Soc Care Community*. 2015;23(3):272-281..

15. Cortinez-O’Ryan A, Albagli A, Sadarangani KP, Aguilar-Farias N. Reclaiming streets for outdoor play: A process and impact evaluation of “Juega en tu Barrio” (Play in your Neighborhood), an intervention to increase physical activity and opportunities for play. *PLoS One*. 2017;12(7).

16. Chapman R, Keall M, Howden-Chapman P, et al. A cost benefit analysis of an active travel intervention with health and carbon emission reduction benefits. *Int J Environ Res Public Health*. 2018;15(5).

17. Jackson G, Thornley S, Woolston J, Papa D, Bernacchi A, Moore T. Reduced acute hospitalisation with the healthy housing programme. *J Epidemiol Community Heal*. 2011;65(7):588-593.

18. Zieff SG, Chaudhuri A, Musselman E. Creating neighborhood recreational space for youth and children in the urban environment: Play(ing in the) Streets in San Francisco. *Child Youth Serv Rev*. 2016;70:95.

19. Beale SJ, Bending MW, Trueman P, Naidoo B. Should we invest in environmental interventions to encourage physical activity in England? An economic appraisal. *Eur J Public Health*. 2012;22(6):869-873.

20. Salt RJ, Costantino ME, Dotson EL, Paper BM. “You Are Not Alone” Strategies for Addressing Mental Health and Health Promotion with a Refugee Women’s Sewing Group. *Issues Ment Health Nurs*. 2017;38(4):337-343.

21. Richardson AS, Ghosh-Dastidar M, Collins RL, et al. Improved Street Walkability, Incivilities, and Esthetics Are Associated with Greater Park Use in Two Low-Income Neighborhoods. *J urban Heal  Bull New York Acad Med*. 2020;27.

22. Jones M, Kimberlee R, Deave T, Evans S. The role of community centre-based arts, leisure and social activities in promoting adult well-being and healthy lifestyles. *Int J Environ Res Public Heal [Electronic Resour*. 2013;10(5):1948-1962.

23. Moulton S, Peck LR, Dillman K-N. Moving to Opportunity’s Impact on Health and Well-Being Among High-Dosage Participants. *Hous Policy Debate*. 2014;24(2):415.

24. Schmitz S. The Impact of Publicly Funded Childcare on Parental Well-Being: Evidence from Cut-Off Rules. *Eur J Popul*. 2019:1-26.

25. Petticrew M, Kearns A, Mason P, Hoy C. The SHARP study: a quantitative and qualitative evaluation of the short-term outcomes of housing and neighbourhood renewal. *BMC Public Health*. 2009;9:415.

26. Fernandez M, Harris B, Becerra M. Examining the complexities of increasing park access in two Latinx neighbourhoods. *Local Environ*. 2019;24(12):1136-1155.

27. Chhabra M, Spector E, Demuynck S, Wiest D, Buckley L, Shea JA. Assessing the relationship between housing and health among medically complex, chronically homeless individuals experiencing frequent hospital use in the United States. *Health Soc Care Community*. 2020;28(1):91-99.

28. Howden-Chapman PL, Keall M, Conlon F, Chapman R. Urban interventions: understanding health co-benefits. *Proc Inst Civ Eng Urban Des Plan*. 2015;168(4):196-203.

29. Egan M, Kearns A, Katikireddi S V, Curl A, Lawson K, Tannahill C. Proportionate universalism in practice? A quasi-experimental study (GoWell) of a UK neighbourhood renewal programme’s impact on health inequalities. *Soc Sci Med*. 2016;152:41-49.

30. Head P, Jackson BE, Bae S, Cherry D. Hospital discharge rates before and after implementation of a city-wide smoking ban in a Texas city, 2004-2008. *Prev Chronic Dis*. 2012;9:E179-E179.

31. Foster S, Hooper P, Knuiman M, Christian H, Bull F, Giles-Corti B. Safe RESIDential Environments? A longitudinal analysis of the influence of crime-related safety on walking. *Int J Behav Nutr Phys Act*. 2016;13:22.

32. Dubbin L, Neufeld S, Kersten E, Yen IH. Health Effects After Renovation (HEAR) Study: Community-Engaged Inquiry Into the Health and Social Impacts of the Rental Assistance Demonstration Program Implementation in San Francisco. *Hous Policy Debate*. 2019;29(3):432-439.

33. Tannis C, Senerat A, Garg M, Peters D, Rajupet S, Garland E. Improving Physical Activity among Residents of Affordable Housing: Is Active Design Enough? *Int J Environ Res Public Heal [Electronic Resour*. 2019;16(1):8.

34. Jongeneel-Grimen B, Droomers M, Kramer D, et al. Impact of a Dutch urban regeneration programme on mental health trends: a quasi-experimental study. *J Epidemiol Community Heal*. 2016;70(10):967-973.

35. Yinon L, Thurston G. An evaluation of the health benefits achieved at the time of an air quality intervention in three Israeli cities. *Environ Int*. 2017;102:66-73.

36. Aldred R, Croft J. Evaluating active travel and health economic impacts of small streetscape schemes: An exploratory study in London. *J Transp Heal*. 2019;12:86-96.

37. Fenelon A, Slopen N, Boudreaux M, Newman SJ. The Impact of Housing Assistance on the Mental Health of Children in the United States. *J Heal Soc Behav*. 2018;59(3):447-463.

38. Novoa AM, Amat J, Malmusi D, et al. Changes in Health Following Housing Improvement in a Vulnerable Population in Spain: A Follow-up Study. *Int J Heal Serv*. 2017;47(1):83-107..

39. Aitken D, Hodgson P, Cook G, Lawson A. Facework and trust in facilitating health-focused housing interventions. *PLoS ONE [Electronic Resour*. 2017;12(4):e0176074.

40. Miller HJ, Tribby CP, Brown BB, et al. Public transit generates new physical activity: Evidence from individual GPS and accelerometer data before and after light rail construction in a neighborhood of Salt Lake City, Utah, USA. *Health Place*. 2015;36:8-17.

41. Bird EL, Ige JO, Pilkington P, Pinto A, Petrokofsky C, Burgess-Allen J. Built and natural environment planning principles for promoting health: an umbrella review. *BMC Public Health*. 2018;18(1):930..

42. Lushey C, et al. Evaluation of the No Wrong Door innovation programme: research report. *Child Soc Care Innov Program Eval Rep*. 2017;(51):75. https://www.gov.uk/government/publications/no-wrong-door-innovation-programme-evaluation.

43. Bekemeier B, Pui-Yan Yip M, Flaxman AD, Barrington W. Five Community-wide Approaches to Physical Activity Promotion: A Cluster Analysis of These Activities in Local Health Jurisdictions in 6 States. *J Public Heal Manag Pract*. 2018;24(2):112-120.

44. Gubbels JS, Kremers SP, Droomers M, et al. The impact of greenery on physical activity and mental health of adolescent and adult residents of deprived neighborhoods: A longitudinal study. *Health Place*. 2016;40:153-160.

45. Fauth RC, Leventhal T, Brooks-Gunn J. Seven Years Later: Effects of a Neighborhood Mobility Program on Poor Black and Latino Adults’ Well-being. *J Health Soc Behav*. 2008;49(2):119-130.

46. Ludwig J, Duncan GJ, Gennetian LA, et al. Neighborhood effects on the long-term well-being of low-income adults. *Science (80- )*. 2012;337(6101):1505-1510. http://www.sciencemag.org/content/337/6101/1505.full.pdf.

47. Shin A, Surkan PJ, Coutinho AJ, et al. Impact of Baltimore Healthy Eating Zones: an environmental intervention to improve diet among African American youth. *Heal Educ Behav*. 2015;42(1):97S-105S.

48. King DM, Jacobson SH. What Is Driving Obesity? A Review on the Connections Between Obesity and Motorized Transportation. *Curr Obes Rep*. 2017;6(1):3-9.

49. Tom Mueller J, Park SY, Mowen AJ. The relationship between self-rated health and local government spending on parks and recreation in the United States from 1997 to 2012. *Prev Med Reports*. 2019;13:105-112.

50. Auchincloss AH, Michael YL, Kuder JF, Shi J, Khan S, Ballester LS. Changes in physical activity after building a greenway in a disadvantaged urban community: A natural experiment. *Prev Med Reports*. 2019;15..

51. Kelaher M, Dunt D, Feldman P, Nolan A, Raban B. The effects of an area-based intervention on the uptake of maternal and child health assessments in Australia: a community trial. *BMC Health Serv Res*. 2009;9:53.

52. Pederson A, Okoli CT, Hemsing N, et al. Smoking on the margins: a comprehensive analysis of a municipal outdoor smoke-free policy. *BMC Public Health*. 2016;16(1):852.

53. Kelaher M, Dunt D, Feldman P, Nolan A, Raban B. The effect of an area-based intervention on breastfeeding rates in Victoria, Australia. *Health Policy (New York)*. 2009;90(1):89-93.

54. Kondo M, Hohl B, Han S, Branas C. Effects of greening and community reuse of vacant lots on crime. *Urban Stud*. 2016;53(15):3279-3295..

55. Ward Thompson C, Curl A, Aspinall P, Alves S, Zuin A. Do changes to the local street environment alter behaviour and quality of life of older adults? The “DIY Streets” intervention. *Br J Sports Med*. 2014;48(13):1059-1065.

56. Thompson SK, Bucerius SM, Luguya M. UNINTENDED CONSEQUENCES OF NEIGHBOURHOOD RESTRUCTURING: Uncertainty, Disrupted Social Networks and In creased Fear of Violent Victimization among Young Adults. *Br J Criminol*. 2013;53(5):924.

57. Reddy AL, Gomez M, Dixon SL. An Evaluation of a State-Funded Healthy Homes Intervention on Asthma Outcomes in Adults and Children. *J Public Heal Manag Pract*. 2017;23(2):219-228.

58. Rissel C, Greaves S, Wen LM, Crane M, Standen C. Use of and short-term impacts of new cycling infrastructure in inner-Sydney, Australia: a quasi-experimental design. *Int J Behav Nutr Phys Act*. 2015;12:129..

59. Vert C, Carrasco-Turigas G, Zijlema W, et al. Impact of a riverside accessibility intervention on use, physical activity, and wellbeing: A mixed methods pre-post evaluation. *Landsc Urban Plan*. 2019;190.

60. Agarwal G, Angeles R, Pirrie M, et al. Effectiveness of a community paramedic-led health assessment and education initiative in a seniors’ residence building: the Community Health Assessment Program through Emergency Medical Services (CHAP-EMS). *BMC Emerg Med*. 2017;17(1):8.

61. Droomers M, Jongeneel-Grimen B, Kramer D, et al. The impact of intervening in green space in Dutch deprived neighbourhoods on physical activity and general health: results from the quasi-experimental URBAN40 study. *J Epidemiol Community Heal*. 2016;70(2):147-154.

62. Kelleher K, Reece J, Sandel M. The Healthy Neighborhood, Healthy Families Initiative. *Pediatrics*. 2018;142(3):9.

63. Edwards P, Steinbach R, Green J, et al. Health impacts of free bus travel for young people: evaluation of a natural experiment in London. *J Epidemiol Community Heal*. 2013;67(8):641-647.

64. Meehan LA, Whitfield GP. Integrating health and transportation in Nashville, Tennessee, USA: From policy to projects. *J Transp Heal*. 2017;4:325-333.

65. Hohl BC, Kondo MC, Kajeepeta S, et al. Creating Safe And Healthy Neighborhoods With Place-Based Violence Interventions. *Health Aff (Millwood)*. 2019;38(10):1687-1694.

66. Prins RG, Panter J, Heinen E, Griffin SJ, Ogilvie DB. Causal pathways linking environmental change with health behaviour change: Natural experimental study of new transport infrastructure and cycling to work. *Prev Med (Baltim)*. 2016;87:175-182.

67. Pérez K, Olabarria M, Rojas-Rueda D, Santamariña-Rubio E, Borrell C, Nieuwenhuijsen M. The health and economic benefits of active transport policies in Barcelona. J Transp Heal. 2017;4:316-324.

68. Kerman N, Aubry T, Adair CE, et al. Effectiveness of Housing First for Homeless Adults with Mental Illness Who Frequently Use Emergency Departments in a Multisite Randomized Controlled Trial. *Adm policy Ment Heal*. 2020;10.

69. Prins RG, Kamphuis CBM, Van Lenthe FJ. The effects of small-scale physical and social environmental interventions on walking behaviour among Dutch older adults living in deprived neighbourhoods: results from the quasi-experimental NEW.ROADS study. *Int J Behav Nutr Phys Act*. 2019;16(1):1-12.

70. Hatchett L, Brown L, Hopkins J, Larsen K, Fournier E. “Something Good Can Grow Here”: Chicago Urban Agriculture Food Projects. *J Prev Interv Community*. 2015;43(2):135-147.

71. South EC, Hohl BC, Kondo MC, MacDonald JM, Branas CC. Effect of Greening Vacant Land on Mental Health of Community-Dwelling Adults: a Cluster Randomized Trial. *JAMA Netw open*. 2018;1(3):e180298.

72. Adar SD, D’Souza J, Sheppard L, et al. Adopting Clean Fuels and Technologies on School Buses. Pollution and Health Impacts in Children. *Am J Respir Crit Care Med*. 2015;191(12):1413-1421.

73. Mari-Dell’Olmo M, Novoa AM, Camprubi L, et al. Housing Policies and Health Inequalities. *Int J Heal Serv*. 2017;47(2):207-232.

74. Thompson CW, Elizalde A, Cummins S, et al. Enhancing health through access to nature: How effective are interventions in woodlands in deprived urban communities? A quasi-experimental study in Scotland, UK. *Sustain*. 2019;11(12).

75. Crawford B, Sainsbury P. Opportunity or Loss? Health Impacts of Estate Renewal and the Relocation of Public Housing Residents. *Urban Policy Res*. 2017;35(2):137-149..

76. Sharpe RA, Machray KE, Fleming LE, et al. Household energy efficiency and health: Area-level analysis of hospital admissions in England. *Environ Int*. 2019;133:105164.

77. Kramer D, Jongeneel-Grimen B, Stronks K, Droomers M, Kunst AE. Are area-based initiatives able to improve area safety in deprived areas? A quasi-experimental evaluation of the Dutch District Approach. *BMC Public Health*. 2015;15:711.

78. Griffiths CJ, Mudway I, Wood H, et al. Impact of the London low emission zone on children’s respiratory health: A sequential yearly cross sectional study 2008-2014. *Thorax*. 2016;71:A182.

79. Frostick C, Watts P, Netuveli G, Renton A, Moore D. Well London: Results of a Community Engagement Approach to Improving Health Among Adolescents from Areas of Deprivation in London. *J Community Pract*. 2017;25(2):235-252.

80. Krieger J, Rabkin J, Sharify D, Song L. High point walking for health: creating built and social environments that support walking in a public housing community. *Am J Public Health*. 2009;99:S593-9.

81. Kramer D, Harting J, Kunst AE. Understanding the impact of area-based interventions on area safety in deprived areas: realist evaluation of a neighbour nuisance intervention in Arnhem, the Netherlands. *BMC Public Health*. 2016;16:291.

82. Thomson K, Hillier-Brown F, Todd A, McNamara C, Huijits T, Bambra C. The effects of public health policies on health inequalities: A review of reviews. *Lancet*. 2017;390:S12.

83. Farrier A, Dooris M, Morley A. Catalysing change? A critical exploration of the impacts of a community food initiative on people, place and prosperity. *Landsc Urban Plan*. 2019;192.

84. Otero I, Nieuwenhuijsen MJ, Rojas-Rueda D. Health impacts of bike sharing systems in Europe. *Environ Int*. 2018;115:387-394.

85. Nelson JD, Wright S, Thomas R, Canning S. The social and economic benefits of community transport in Scotland. *Case Stud Transp Policy*. 2017;5(2):286-298.

86. Kondo MC, Morrison C, Jacoby SF, et al. Blight Abatement of Vacant Land and Crime in New Orleans. *Public Health Rep*. 2018;133(6):650-657.

87. Bennett E, et al. Warm, safe and well: the evaluation of the Warm at Home programme. 2016:vii, 74. http://filt.org.uk/wp-content/uploads/2016/11/Warm-at-Home-report.pdf.

88. Malden S, Jepson R, Laird Y, McAteer J. A theory based evaluation of an intervention to promote positive health behaviors and reduce social isolation in people experiencing homelessness. *J Soc Distress Homeless*. 2019;28(2):158-168.

89. Coventry PA, Neale C, Dyke A, Pateman R, Cinderby S. The Mental Health Benefits of Purposeful Activities in Public Green Spaces in Urban and Semi-Urban Neighbourhoods: A Mixed-Methods Pilot and Proof of Concept Study. *Int J Environ Res Public Heal [Electronic Resour*. 2019;16(15):30.

90. Sami M, Smith M, Ogunseitan OA. Changes in Physical Activity After Installation of a Fitness Zone in a Community Park. *Prev Chronic Dis*. 2018;15:E101.

91. Bond L, Kearns A, Mason P, Tannahill C, Egan M, Whitely E. Exploring the relationships between housing, neighbourhoods and mental wellbeing for residents of deprived areas. *BMC Public Health*. 2012;12:48.

92. Prins RG, Foley L, Mutrie N, Ogilvie DB. Effects of urban motorways on physical activity and sedentary behaviour in local residents: A natural experimental study. *Int J Behav Nutr Phys Act*. 2017;14(1). http://www.ijbnpa.org/home/.

93. Bray N, Burns P, Jones A, Winrow E, Edwards RT. Costs and outcomes of improving population health through better social housing: a cohort study and economic analysis. *Int J Public Health*. 2017;62(9):1039-1050.

94. Nightingale CM, Limb ES, Ram B, et al. The effect of moving to East Village, the former London 2012 Olympic and Paralympic Games Athletes’ Village, on physical activity and adiposity (ENABLE London): a cohort study. *Lancet Public Heal*. 2019;4(8):e421-e430. http://thelancet.com/journals/lanpub/issue/vol1no2/PIIS2468-2667(16)X0003-5.

95. Nicholls L, Phelan K, Maller C. ‘A Fantasy to Get Employment Around the Area’: Long Commutes and Resident Health in an Outer Urban Master-Planned Estate. *Urban Policy Res*. 2018;36(1):48-62.

96. Ruijsbroek A, Wong A, Kunst AE, et al. The impact of urban regeneration programmes on health and health-related behaviour: Evaluation of the Dutch District Approach 6.5 years from the start. *PLoS ONE [Electronic Resour*. 2017;12(5):e0177262.

97. Keall M, Chapman R, Howden-Chapman P, Witten K, Abrahamse W, Woodward A. Increasing active travel: results of a quasi-experimental study of an intervention to encourage walking and cycling. *J Epidemiol Community Heal*. 2015;69(12):1184-1190.

98. Egan M, Lawson L, Kearns A, Conway E, Neary J. Neighbourhood demolition, relocation and health. A qualitative longitudinal study of housing-led urban regeneration in Glasgow, UK. *Health Place*. 2015;33:101-108.

99. Panter J, Ogilvie D, iConnect consortium. Theorising and testing environmental pathways to behaviour change: natural experimental study of the perception and use of new infrastructure to promote walking and cycling in local communities. *BMJ Open*. 2015;5(9):e007593.

100. Taylor J, Cole R, Kynn M, Lowe J. Home away from home: Health and wellbeing benefits of men’s sheds. *Heal Promot J Aust*. 2018;29(3):236-242.

101. Baybutt M, Dooris M, Farrier A. Growing health in UK prison settings. *Health Promot Int*. 2019;34(4):792-802.

102. Ellen IG, Dragan KL, Glied S. Renovating Subsidized Housing: The Impact On Tenants’ Health. *Health Aff (Millwood)*. 2020;39(2):224-232.

103. Hurt RD, Weston SA, Ebbert JO, et al. Myocardial infarction and sudden cardiac death in Olmsted County, Minnesota, before and after smoke-free workplace laws. *Arch Intern Med*. 2012;172(21):1635-1641.

104. Menec VH, Nowicki S. Examining the relationship between communities’ “age-friendliness” and life satisfaction and self-perceived health in rural Manitoba, Canada. *Rural Remote Heal*. 2014;14:2594.

105. Schultz CL, Wilhelm Stanis SA, Sayers SP, Thombs LA, Thomas IM. A longitudinal examination of improved access on park use and physical activity in a low-income and majority African American neighborhood park. *Prev Med (Baltim)*. 2017;95:S95-S100.

106. Webb MD, Rohe WM, Nguyen MT, Frescoln K, Donegan M, Han HS. Finding HOPE: Changes in depressive symptomology following relocation from distressed public housing. *Soc Sci Med*. 2017;190:165-173.

107. Richardson AS, Ghosh-Dastidar M, Beckman R, et al. Can the introduction of a full-service supermarket in a food desert improve residents’ economic status and health? *Ann Epidemiol*. 2017;27(12):771-776.

108. Clark A. How can local authorities with less money support better outcomes for older people? *(Solutions; January 2011*. 2011:15p., bibliog. http://www.jrf.org.uk/sites/default/files/jrf/migrated/files/authorities-supporting-older-people-summary.pdf.

109. Serrano E, Larranaga I, Morteruel M, et al. Urban regeneration as population health intervention: a health impact assessment in the Bay of Pasaia (Spain). *Int J Equity Health*. 2016;15(1):145.

110. Liao Y, Siegel PZ, Garraza LG, et al. Reduced Prevalence of Obesity in 14 Disadvantaged Black Communities in the United States: A Successful 4-Year Place-Based Participatory Intervention. *Am J Public Health*. 2016;106(8):1442-1448.

111. Ayres L, Patrick R, Capetola T. Health and environmental impacts of a regional Australian Men’s Shed program. *Aust J Rural Health*. 2018;26(1):65-67.

112. Mattocks N, Meyer M, Hopkins KM, Cohen-Callow A. Clean and green organizing in urban neighborhoods: Measuring perceived and objective outcomes. *J Community Pract*. 2019;27(3):351-368.

113. Ram B, Nightingale CM, Hudda MT, et al. Cohort profile: Examining Neighbourhood Activities in Built Living Environments in London: the ENABLE London-Olympic Park cohort. *BMJ Open*. 2016;6(10):e012643.

114. Bowen DJ, Quintiliani LM, Bhosrekar SG, Goodman R, Smith E. Changing the housing environment to reduce obesity in public housing residents: a cluster randomized trial. *BMC Public Health*. 2018;18(1):883.

115. Lopez PM, Islam N, Feinberg A, et al. A Place-Based Community Health Worker Program: Feasibility and Early Outcomes, New York City, 2015. *Am J Prev Med*. 2017;52(3):S284-S289.

116. Wright L, Peasgood T. Housing for vulnerable people: cost-effectiveness analysis of Housing First. 2018:22. https://whatworkswellbeing.org/product/housing-for-vulnerable-people-full-report/.

117. Egan M, Katikireddi S V, Kearns A, Tannahill C, Kalacs M, Bond L. Health effects of neighborhood demolition and housing improvement: a prospective controlled study of 2 natural experiments in urban renewal. *Am J Public Health*. 2013;103(6):e47-53.

118. Roe J, Barnes L, Napoli NJ, Thibodeaux J. The restorative health benefits of a tactical urban intervention: An urban waterfront study. *Front Built Environ*. 2019;5. https://www.scopus.com/inward/record.uri?eid=2-s2.0-

119. Lim S, Singh TP, Hall G, Walters S, Gould LH. Impact of a New York City Supportive Housing Program on Housing Stability and Preventable Health Care among Homeless Families. *Health Serv Res*. 2018;53(5):3437-3454.

120. Murage P, Hajat S, Bone A. Variation in Cold-Related Mortality in England Since the Introduction of the Cold Weather Plan: Which Areas Have the Greatest Unmet Needs? *Int J Environ Res Public Heal [Electronic Resour*. 2018;15(11):19.

121. Ward Thompson C, Roe J, Aspinall P. Woodland improvements in deprived urban communities: What impact do they have on people’s activities and quality of life? *Landsc Urban Plan*. 2013;118:79-89.

122. Kim S, Zafari Z, Bellanger M, Muennig PA. Cost-Effectiveness of Capping Freeways for Use as Parks: The New York Cross-Bronx Expressway Case Study. *Am J Public Health*. 2018;108(3):379-384.

123. Panter J, Ogilvie D, iConnect consortium. Can environmental improvement change the population distribution of walking? *J Epidemiol Community Heal*. 2017;71(6):528-535.

124. Smith BJ, MacKenzie-Stewart R, Newton FJ, et al. A longitudinal study examining uptake of new recreation infrastructure by inactive adults. *Int J Behav Nutr Phys Act*. 2019;16(1)

125. Dockery DW, Rich DQ, Goodman PG, et al. Effect of air pollution control on mortality and hospital admissions in Ireland. *Res Rep - Heal Eff Inst*. 2013;(176):3-109.

126. MC K, Andreyeva E, EC S, JM M, CC B. Neighborhood Interventions to Reduce Violence. *Annu Rev Public Health*. 2018;39:253-271. https://pubmed.ncbi.nlm.nih.gov/29328874/.

***Intervention implemented pre-2008 (n=101)***

1. Farrelly MC, Loomis BR, Kuiper N, et al. Are tobacco control policies effective in reducing young adult smoking? *J Adolesc Heal*. 2014;54(4):481-486.

2. Greene G, Fone D, Farewell D, et al. Improving mental health through neighbourhood regeneration: the role of cohesion, belonging, quality and disorder. *Eur J public Heal*. 2019;10.

3. Wolitski RJ, Kidder DP, Pals SL, et al. Randomized trial of the effects of housing assistance on the health and risk behaviors of homeless and unstably housed people living with HIV. *AIDS Behav*. 2010;14(3):493-503.

4. Brown V, Diomedi BZ, Moodie M, Veerman JL, Carter R. A systematic review of economic analyses of active transport interventions that include physical activity benefits. *Transp Policy*. 2016;45:190-208.

5. Johnson R. Pervasive interactions: a purposive best evidence review with methodological observations on the impact of housing circumstances and housing interventions on adult mental health and well-being. *Housing, Care Support*. 2013;16(1):32-49.

6. Roe B, Beech R, Harris M, et al. Improving quality of life for older people in the community: findings from a local Partnerships for Older People Project innovation and evaluation. *Prim Health Care Res Dev*. 2011;12(3):200-213.

7. Dixon SL, Fowler C, Harris J, et al. An examination of interventions to reduce respiratory health and injury hazards in homes of low-income families. *Environ Res*. 2009;109(1):123-130.

8. Zablocki RW, Edland SD, Myers MG, Strong DR, Hofstetter CR, Al-Delaimy WK. Smoking ban policies and their influence on smoking behaviors among current California smokers: a population-based study. *Prev Med (Baltim)*. 2014;59:73-78.

9. Pollack CE, Du S, Blackford AL, Herring B. Experiment to decrease neighborhood poverty had limited effects on emergency department use. *Health Aff*. 2019;38(9):1442-1450.

10. Boelsen-Robinson T, Peeters A, Beauchamp A, Chung A, Gearon E, Backholer K. A systematic review of the effectiveness of whole-of-community interventions by socioeconomic position. *Obes Rev*. 2015;16(9):806-816.

11. Fitzpatrick-Lewis D, Ganann R, Krishnaratne S, Ciliska D, Kouyoumdjian F, Hwang SW. Effectiveness of interventions to improve the health and housing status of homeless people: a rapid systematic review. *BMC Public Health*. 2011;11:638.

12. Kearns A, Ghosh S, Mason P, Egan M. Urban regeneration and mental health: Investigating the effects of an area-based intervention using a modified intention to treat analysis with alternative outcome measures. *Heal Place*. 2020..

13. Spencer AJ, Armfield JM, Slade GD. Exposure to water fluoridation and caries increment. *Community Dent Health*. 2008;25(1):12-22.

14. Chapman R, Howden-Chapman P, Viggers H, O’Dea D, Kennedy M. Retrofitting houses with insulation: a cost-benefit analysis of a randomised community trial. *J Epidemiol Community Heal*. 2009;63(4):271-277.

15. Cohen DA, Marsh T, Williamson S, et al. The potential for pocket parks to increase physical activity. *Am J Heal Promot*. 2014;28(3):S19-26.

16. Droomers M, Jongeneel-Grimen B, Bruggink JW, Kunst A, Stronks K. Is it better to invest in place or people to maximize population health? Evaluation of the general health impact of urban regeneration in Dutch deprived neighbourhoods. *Health Place*. 2016;41:50-57.

17. Rosenblatt P, DeLuca S. What Happened in Sandtown-Winchester? Understanding the Impacts of a Comprehensive Community Initiative. *Urban Aff Rev*. 2017;53(3):463-494.

18. Dulin-Keita A, Clay O, Whittaker S, et al. The influence of HOPE VI neighborhood revitalization on neighborhood-based physical activity: A mixed-methods approach. Soc Sci Med. 2015;139:90-99.

19. Lindberg RA, Shenassa ED, Acevedo-Garcia D, Popkin SJ, Villaveces A, Morley RL. Housing interventions at the neighborhood level and health: a review of the evidence. *J Public Heal Manag Pract*. 2010;16(5):S44-52.

20. Kondo MC, South EC, Branas CC. Nature-Based Strategies for Improving Urban Health and Safety. *J Urban Heal*. 2015;92(5):800-814.

21. Gustat J, Rice J, Parker KM, Becker AB, Farley TA. Effect of changes to the neighborhood built environment on physical activity in a low-income African American neighborhood. *Prev Chronic Dis*. 2012;9:E57.

22. Thomson H, Jepson R, Hurley F, Douglas M. Assessing the unintended health impacts of road transport policies and interventions: translating research evidence for use in policy and practice. *BMC Public Health*. 2008;8:339.

23. Lorenc T, Petticrew M, Welch V, Tugwell P. What types of interventions generate inequalities? Evidence from systematic reviews. *J Epidemiol Community Heal*. 2013;67(2):190-193.

24. Nguyen Q, Schmidt N, Tchetgen E, Glymour M, Almeida J, Osypuk TL. Effects of a housing mobility experiment on neighborhood quality. *Am J Epidemiol*. 2013;177:S83.

25. Hult M, Lappalainen K, Saaranen TK, Räsänen K, Vanroelen C, Burdorf A. Health‐improving interventions for obtaining employment in unemployed job seekers. *Cochrane Database Syst Rev*. 2020;(1). http://dx.doi.org/10.1002/14651858.CD013152.pub2.

26. Beatty C, et al. New deal for communities: a synthesis of new programme wide evidence: 2006-07: NDC national evaluation phase 2. *Res Rep*. 2008;(39):86p.

27. Bambra C, Gibson M, Sowden A, Wright K, Whitehead M, Petticrew M. Tackling the wider social determinants of health and health inequalities: Evidence from systematic reviews. *J Epidemiol Community Health*. 2010. doi:10.1136/jech.2008.082743

28. Leventhal T, Dupere V. Moving to Opportunity: Does long-term exposure to “low-poverty” neighborhoods make a difference for adolescents. *Soc Sci Med*. 2011;73(5):737-743.

29. Wilson NJ, Cordier R. A narrative review of Men’s Sheds literature: reducing social isolation and promoting men’s health and well-being. *Health Soc Care Community*. 2013;21(5):451-463.

30. Yang L, Sahlqvist S, McMinn A, Griffin SJ, Ogilvie D. Interventions to promote cycling: systematic review. *BMJ*. 2010;341:c5293.

31. Johansson P, Sadigh S, Tillgren P, Rehnberg C. Non-pharmaceutical prevention of hip fractures - A cost-effectiveness analysis of a community-based elderly safety promotion program in Sweden. *Cost Eff Resour Alloc*. 2008;6.

32. White J, Greene G, Farewell D, et al. Improving Mental Health Through the Regeneration of Deprived Neighborhoods: A Natural Experiment. *Am J Epidemiol*. 2017;186(4):473-480.

33. Kramer D, Lakerveld J, Stronks K, Kunst AE. Uncovering How Urban Regeneration Programs May Stimulate Leisure-time Walking Among Adults in Deprived Areas: A Realist Review. *Int J Heal Serv*. 2017;47(4):703-724.

34. Kelaher M, Warr DJ, Tacticos T. Evaluating health impacts: Results from the neighbourhood renewal strategy [corrected] in Victoria, Australia. *Health Place*. 2010;16(5):861-867.

35. Hayes SL, Mann MK, Morgan FM, Kelly MJ, Weightman AL. Collaboration between local health and local government agencies for health improvement. *Cochrane Database Syst Rev*. 2012;10:CD007825.

36. Mehdipanah R, Malmusi D, Muntaner C, Borrell C. An evaluation of an urban renewal program and its effects on neighborhood resident’s overall wellbeing using concept mapping. *Health Place*. 2013;23:9-17.

37. Zapata Moya AR, Navarro Yanez CJ. Impact of area regeneration policies: performing integral interventions, changing opportunity structures and reducing health inequalities. *J Epidemiol Community Heal*. 2017;71(3):239-247.

38. Howden-Chapman P, Crane J, Chapman R, Fougere G. Improving health and energy efficiency through community-based housing interventions. *Int J Public Health*. 2011;56(6):583-588.

39. Chomitz VR, McGowan RJ, Wendel JM, et al. Healthy Living Cambridge Kids: a community-based participatory effort to promote healthy weight and fitness. *Obesity*. 2010;18:S45-53.

40. Pollack CE, Blackford AL, Du S, Deluca S, Thornton RLJ, Herring B. Association of Receipt of a Housing Voucher with Subsequent Hospital Utilization and Spending. *JAMA - J Am Med Assoc*. 2019;322(21):2115-2124.

41. Lovell R, Husk K, Cooper C, Stahl-Timmins W, Garside R. Understanding how environmental enhancement and conservation activities may benefit health and wellbeing: a systematic review. *BMC Public Health*. 2015;15:864.

42. Schnake-Mahl AS, Jahn JL, Subramanian S V, Waters MC, Arcaya M. Gentrification, Neighborhood Change, and Population Health: a Systematic Review. *J Urban Health*. 2020;97(1):1-25.

43. Williams O. Identifying adverse effects of area-based health policy: An ethnographic study of a deprived neighbourhood in England. *Health Place*. 2017;45:85-91..

44. Main C, Thomas S, Ogilvie D, et al. Population tobacco control interventions and their effects on social inequalities in smoking: placing an equity lens on existing systematic reviews. *BMC Public Health*. 2008;8:178.

45. Mehdipanah R, Rodriguez-Sanz M, Malmusi D, et al. The effects of an urban renewal project on health and health inequalities: a quasi-experimental study in Barcelona. *J Epidemiol Community Heal*. 2014;68(9):811-817.

46. Willand N, Ridley I, Maller C. Towards explaining the health impacts of residential energy efficiency interventions - A realist review. Part 1: Pathways. *Soc Sci Med*. 2015;133:191-201.

47. Solomon EM, Wing H, Steiner JF, Gottlieb LM. Impact of Transportation Interventions on Health Care Outcomes: A Systematic Review. *Med Care*. 2020..

48. Hahn EJ, Rayens MK, Burkhart P V, Moser DK. Smoke-free laws, gender, and reduction in hospitalizations for acute myocardial infarction. *Public Health Rep*. 2011;126(6):826-833.

49. Stafford M, Badland H, Nazroo J, et al. Evaluating the health inequalities impact of area-based initiatives across the socioeconomic spectrum: a controlled intervention study of the New Deal for Communities, 2002-2008. *J Epidemiol Community Heal*. 2014;68(10):979-986.

50. Cairns J, Warren J, Garthwaite K, Greig G, Bambra C. Go slow: An umbrella review of the effects of 20 mph zones and limits on health and health inequalities. *J Public Heal (United Kingdom)*. 2015. doi:10.1093/pubmed/fdu067

51. Rigby E, Hatch ME. Incorporating Economic Policy Into A “Health-In-All-Policies” Agenda. *Health Aff*. 2016;35(11):2044-2052.

52. Howden-Chapman P, Keall M, Whitwell K, Chapman R. Evaluating natural experiments to measure the co-benefits of urban policy interventions to reduce carbon emissions in New Zealand. *Sci Total Environ*. 2020;700.

53. Townshend T, Lake A. Obesogenic environments: Current evidence of the built and food environments. *Perspect Public Health*. 2016;137(1):38-44.

54. Graif C, Arcaya MC, Diez Roux A V. Moving to opportunity and mental health: Exploring the spatial context of neighborhood effects. *Soc Sci Med*. 2016;162:50-58.

55. Hooper P, Giles-Corti B, Knuiman M. Evaluating the implementation and active living impacts of a state government planning policy designed to create walkable neighborhoods in Perth, Western Australia. *Am J Heal Promot*. 2014;28:S5-S18.

56. Cesaroni G, Boogaard H, Jonkers S, et al. Health benefits of traffic-related air pollution reduction in different socioeconomic groups: the effect of low-emission zoning in Rome. *Occup Environ Med*. 2012;69(2):133-139.

57. Lorenc T, Petticrew M, Whitehead M, et al. Environmental interventions to reduce fear of crime: systematic review of effectiveness. *Syst Rev*. 2013;2:30.

58. Osypuk TL, Tchetgen Tchetgen EJ, Acevedo-Garcia D, et al. Differential mental health effects of neighborhood relocation among youth in vulnerable families: Results from a randomized trial. *Arch Gen Psychiatry*. 2012;69(12):1284-1294.

59. Henneman LRF, Choirat C, Zigler CM. Accountability Assessment of Health Improvements in the United States Associated with Reduced Coal Emissions between 2005 and 2012. *Epidemiology*. 2019;30(4):477-485..

60. Liu Y, Yan Z, Dong C. Health implications of improved air quality from Beijing’s driving restriction policy. *Environ Pollut*. 2016;219:323-328.

61. Coffield E, Nihiser AJ, Sherry B, Economos CD. Shape Up Somerville: change in parent body mass indexes during a child-targeted, community-based environmental change intervention. *Am J Public Health*. 2015;105(2):e83-9.

62. Michael YL, Nagel CL, Gold R, Hillier TA. Does change in the neighborhood environment prevent obesity in older women? Soc Sci Med. 2014;102:129-137.

63. de Nazelle A, Nieuwenhuijsen MJ, Anto JM, et al. Improving health through policies that promote active travel: a review of evidence to support integrated health impact assessment. *Environ Int*. 2011;37(4):766-777.

64. Muennig P, Masters R, Vail D, Hakes J. The effects of New York City’s coordinated public health programmes on mortality through 2011. *Int J Epidemiol*. 2017;46(4):1239-1248.

65. Stewart G, Anokye NK, Pokhrel S. What interventions increase commuter cycling? A systematic review. *BMJ Open*. 2015;5(8):e007945.

66. Shaw C, Hales S, Howden-Chapman P, Edwards R. Health co-benefits of climate change mitigation policies in the transport sector. *Nat Clim Chang*. 2014;4(6):427-433..

67. Osypuk TL, Schmidt NM, Kehm RD, Tchetgen Tchetgen EJ, Glymour MM. The price of admission: does moving to a low-poverty neighborhood increase discriminatory experiences and influence mental health? *Soc Psychiatry Psychiatr Epidemiol*. 2019;54(2):181-190.

68. Gibson M, Thomson H, Kearns A, Petticrew M. Understanding the psychosocial impacts of housing type: Qualitative evidence from a housing and regeneration intervention. *Hous Stud*. 2011;26(4):555-573.

69. Tokarchuk O, Gabriele R, Maurer O. Development of city tourism and well-being of urban residents: A case of German Magic Cities. *Tour Econ*. 2017;23(2):343.

70. Hooper P, Foster S, Bull F, et al. Living liveable? RESIDE’s evaluation of the “Liveable Neighborhoods” planning policy on the health supportive behaviors and wellbeing of residents in Perth, Western Australia. *SSM - Popul Heal*. 2020;10.

71. Melhuish E, Belsky J, Leyland AH, Barnes J, of Sure Start Research T. Effects of fully-established Sure Start Local Programmes on 3-year-old children and their families living in England: a quasi-experimental observational study. *Lancet*. 2008;372(9650):1641-1647.

72. Baba C, Kearns A, McIntosh E, Tannahill C, Lewsey J. Is empowerment a route to improving mental health and wellbeing in an urban regeneration (UR) context? *Urban Stud*. 2017. doi:10.1177/0042098016632435

73. El Ansari W, El-Silimy S. Are fuel poverty reduction schemes associated with decreased excess winter mortality in elders? A case study from London, U.K. *Chronic Illn*. 2008;4(4):289-294.

74. Stafford M, Nazroo J, Popay JM, Whitehead M. Tackling inequalities in health: evaluating the New Deal for Communities initiative. *J Epidemiol Community Heal*. 2008;62(4):298-304.

75. Jensen JA, Schillo BA, Moilanen MM, et al. Tobacco smoke exposure in nonsmoking hospitality workers before and after a state smoking ban. *Cancer Epidemiol Biomarkers Prev*. 2010;19(4):1016-1021.

76. Nguyen QC, Schmidt NM, Glymour MM, Rehkopf DH, Osypuk TL. Were the mental health benefits of a housing mobility intervention larger for adolescents in higher socioeconomic status families? *Health Place*. 2013;23:79-88.

77. Nickel S, von dem Knesebeck O. Effectiveness of Community-Based Health Promotion Interventions in Urban Areas: A Systematic Review. *J community Heal*. 2019;11.

78. Komro KA, Tobler AL, Delisle AL, O’Mara RJ, Wagenaar AC. Beyond the clinic: improving child health through evidence-based community development. *BMC Pediatr*. 2013;13:172.

79. Lipek T, Igel U, Gausche R, Kiess W, Grande G. Obesogenic environments: environmental approaches to obesity prevention. *J Pediatr Endocrinol Metab*. 2015;28(5):485-495.

80. Tester J, Baker R. Making the playfields even: evaluating the impact of an environmental intervention on park use and physical activity. *Prev Med (Baltim)*. 2009;48(4):316-320.

81. Jacobs DE, Brown MJ, Baeder A, et al. A systematic review of housing interventions and health: introduction, methods, and summary findings. *J Public Heal Manag Pract*. 2010;16(5):S5-10.

82. Thomas S, Fayter D, Misso K, et al. Population tobacco control interventions and their effects on social inequalities in smoking: systematic review. *Tob Control*. 2008;17(4):230-237.

83. Castro A, Kunzli N, Gotschi T. Health benefits of a reduction of PM_10_ and NO_2_ exposure after implementing a clean air plan in the Agglomeration Lausanne-Morges. *Int J Hyg Environ Heal*. 2017;220(5):829-839.

84. Jackson L, Langille L, Lyons R, Hughes J, Martin D, Winstanley V. Does moving from a high-poverty to lower-poverty neighborhood improve mental health? A realist review of “Moving to Opportunity.” *Heal Place*. 2009;15(4):961-970..

85. Maidment CD, Jones CR, Webb TL, Hathway EA, Gilbertson JM. The impact of household energy efficiency measures on health: a meta-analysis. *Energy Policy*. 2014;65:583-593.

86. Gibson M, Petticrew M, Bambra C, Sowden AJ, Wright KE, Whitehead M. Housing and health inequalities: A synthesis of systematic reviews of interventions aimed at different pathways linking housing and health. *Heal Place*. 2011. doi:10.1016/j.healthplace.2010.09.011

87. Rogers A, Huxley P, Evans S, Gately C. More than jobs and houses: mental health, quality of life and the perceptions of locality in an area undergoing urban regeneration. *Soc Psychiatry Psychiatr Epidemiol*. 2008;43(5):364-372.

88. Pucher J, Dill J, Handy S. Infrastructure, programs, and policies to increase bicycling: an international review. *Prev Med (Baltim)*. 2010;50:S106-25..

89. Poortinga W, Jones N, Lannon S, Jenkins H. Social and health outcomes following upgrades to a national housing standard: a multilevel analysis of a five-wave repeated cross-sectional survey. *BMC Public Health*. 2017;17(1):927.

90. Hopkins DP, Razi S, Leeks KD, Priya Kalra G, Chattopadhyay SK, Soler RE. Smokefree policies to reduce tobacco use a systematic review. *Am J Prev Med*. 2010;38:S275-89.

91. Corburn J, Curl S, Arredondo G. A health-in-all-policies approach addresses many of Richmond, California’s place-based hazards, stressors. *Health Aff*. 2014;33(11):1905-1913.

92. Meyer MRU, Perry CK, Sumrall JC, et al. Physical Activity-Related Policy and Environmental Strategies to Prevent Obesity in Rural Communities: A Systematic Review of the Literature, 2002-2013. *Prev Chronic Dis*. 2016;13:1-24.

93. Moffa M, Cronk R, Fejfar D, Dancausse S, Padilla LA, Bartram J. A systematic scoping review of environmental health conditions and hygiene behaviors in homeless shelters. *Int J Hyg Environ Heal*. 2019;222(3):335-346.

94. Moffa M, Cronk R, Fejfar D, Dancausse S, Padilla LA, Bartram J. A systematic scoping review of hygiene behaviors and environmental health conditions in institutional care settings for orphaned and abandoned children. *Sci Total Environ*. 2019;658:1161-1174.

95. Park ES, Sener IN. Impact of light rail transit on traffic-related pollution and stroke mortality. *Int J Public Health*. 2017;62(7):721-728.

96. Beauchamp A, Backholer K, Magliano D, Peeters A. The effect of obesity prevention interventions according to socioeconomic position: a systematic review. *Obes Rev*. 2014;15(7):541-554.

97. Gibson M, et al. Welfare-to-work interventions and their effects on the mental and physical health of lone parents and their children. *Cochrane Database Syst Rev*. 2018;(2):113.

98. Foster S, Hooper P, Knuiman M, Bull F, Giles-Corti B. Are liveable neighbourhoods safer neighbourhoods? Testing the rhetoric on new urbanism and safety from crime in Perth, Western Australia. *Soc Sci Med*. 2016;164:150-157.

99. Hahn EJ. Smokefree legislation: a review of health and economic outcomes research. *Am J Prev Med*. 2010;39(6):S66-76.

100. Batty E, et al. The New Deal for Communities experience: a final assessment: the New Deal for Communities evaluation: final report: volume 7. 2010:45p.

101. Stevens M, Roberts H, Shiell A. Research review: economic evidence for interventions in children’s social care: revisiting the What Works for Children project. *Child Fam Soc Work*. 2010;15(2):145-154..

***No intervention/exploratory studies (n=67)***

1. Stone GA, Fernandez M, DeSantiago A. Rural Latino health and the built environment: a systematic review. *Ethn Health*. 2019:1-26.

2. Lund C, Brooke-Sumner C, Baingana F, et al. Social determinants of mental disorders and the Sustainable Development Goals: a systematic review of reviews. *The Lancet Psychiatry*. 2018;5(4):357-369.

3. Gibbons J, Barton M, Brault E. Evaluating gentrification’s relation to neighborhood and city health. *PLoS One*. 2018;13(11).

4. Mueller N, Rojas-Rueda D, Cirach M, Martinez D, Nieuwenhuijsen M. Health impact assessment of barcelonas superblock model. *Occup Environ Med*. 2018;75:A38-A39.

5. Pastor M, Morello-Frosch R. Integrating public health and community development to tackle neighborhood distress and promote well-being. *Health Aff*. 2014;33(11):1890-1896.

6. Tosi MC, Turvani ME, Munarin S. Public realm as city welfare &amp; citizens wellbeing: the case of Cao Yang–Shanghai. *J Archit Urban*. 2017;41(2):101-109.

7. Tran LD, Rice TH, Ong PM, Banerjee S, Liou J, Ponce NA. Impact of gentrification on adult mental health. *Heal Serv Res*. 2020. http://onlinelibrary.wiley.com/journal/10.1111/(ISSN)1475-6773.

8. Nieuwenhuijsen MJ, Khreis H. Car free cities: Pathway to healthy urban living. *Environ Int*. 2016;94:251-262.

9. Barnett DW, Barnett A, Nathan A, et al. Built environmental correlates of older adults’ total physical activity and walking: a systematic review and meta-analysis. *Int J Behav Nutr Phys Act*. 2017;14(1):103.

10. Xu F, Jin L, Qin Z, et al. Access to public transport and childhood obesity: A systematic review. *Obes Rev*. 2020. http://onlinelibrary.wiley.com/journal/10.1111/(ISSN)1467-789X.

11. Lazareva O. The effect of labor market shocks on health: The case of the Russian transition. *Econ Hum Biol*. 2020;36.

12. Gotschi T. Costs and benefits of bicycling investments in Portland, Oregon. *J Phys Act Health*. 2011;8:S49-58.

13. Carlin A, Perchoux C, Puggina A, et al. A life course examination of the physical environmental determinants of physical activity behaviour: A “Determinants of Diet and Physical Activity” (DEDIPAC) umbrella systematic literature review. PLoS One. 2017;12(8).

14. Mueller N, Rojas-Rueda D, Cole-Hunter T, et al. Health impact assessment of active transportation: A systematic review. *Prev Med (Baltim)*. 2015;76:103-114.

15. Ottoni CA, Sims-Gould J, Winters M, Heijnen M, McKay H. “Benches become like porches”: Built and social environment influences on older adults’ experiences of mobility and well-being. *Soc Sci Med*. 2016;169:33.

16. Mattheys K, Warren J, Bambra C. “Treading in sand”: a qualitative study of the impact of austerity on inequalities in mental health. *Soc Policy Adm*. 2018;52(7):1275-1289. http://onlinelibrary.wiley.com/journal/10.1111/(ISSN)1467-9515.

17. Mueller N, Rojas-Rueda D, Khreis H, et al. Socioeconomic inequalities in urban and transport planning related exposures and mortality: A health impact assessment study for Bradford, UK. *Environ Int*. 2018;121:931-941.

18. Smith RJ, Lehning AJ, Kim K. Aging in Place in Gentrifying Neighborhoods: Implications for Physical and Mental Health. *Gerontologist*. 2018;58(1):26-35.

19. Nordbo ECA, Nordh H, Raanaas RK, Aamodt G. Promoting activity participation and well-being among children and adolescents: a systematic review of neighborhood built-environment determinants. *JBI database Syst Rev Implement reports*. 2019;30.

20. Brown V, Moodie M, Mantilla Herrera AM, Veerman JL, Carter R. Active transport and obesity prevention - A transportation sector obesity impact scoping review and assessment for Melbourne, Australia. *Prev Med (Baltim)*. 2017;96:49-66.

21. Adams J, Witten K, Conway K. Community development as health promotion: evaluating a complex locality-based project in New Zealand. *Community Dev J*. 2009;44(2):140-157.

22. Larson LR, Jennings V, Cloutier SA. Public Parks and Wellbeing in Urban Areas of the United States. *PLoS ONE [Electronic Resour*. 2016;11(4):e0153211.

23. Langlois M, Wasfi RA, Ross NA, El-Geneidy AM. Can transit-oriented developments help achieve the recommended weekly level of physical activity? *J Transp Heal*. 2016;3(2):181-190.

24. Stevenson M, Thompson J, de Sa TH, et al. Land use, transport, and population health: estimating the health benefits of compact cities. *Lancet*. 2016;388(10062):2925-2935.

25. Aguilar-Palacio I, Carrera-Lasfuentes P, Sanchez-Recio R, Alonso JP, Rabanaque MJ. Recession, employment and self-rated health: a study on the gender gap. *Public Health*. 2018;154:44-50..

26. Douglas JA, Briones MD, Bauer EZ, Trujillo M, Lopez M, Subica AM. Social and environmental determinants of physical activity in urban parks: Testing a neighborhood disorder model. *Prev Med (Baltim)*. 2018;109:119-124. http://www.elsevier.com/inca/publications/store/6/2/2/9/3/4/index.htt.

27. Zenk SN, Tarlov E, Wing C, et al. Geographic Accessibility Of Food Outlets Not Associated With Body Mass Index Change Among Veterans, 2009-14. *Health Aff*. 2017;36(8):1433-1442.

28. Sykes KE, Robinson KN. Making the right moves: promoting smart growth and active aging in communities. *J Aging Soc Policy*. 2014;26(1):166-180.

29. Bentley R, Blakely T, Kavanagh A, et al. A Longitudinal Study Examining Changes in Street Connectivity, Land Use, and Density of Dwellings and Walking for Transport in Brisbane, Australia. *Environ Health Perspect*. 2018;126(5):57003..

30. Yang L, Griffin S, Khaw KT, Wareham N, Panter J. Longitudinal associations between built environment characteristics and changes in active commuting. *BMC Public Health*. 2017;17(1):458.

31. Nightingale CM, Rudnicka AR, Ram B, et al. Housing, neighbourhood and sociodemographic associations with adult levels of physical activity and adiposity: baseline findings from the ENABLE London study. *BMJ Open*. 2018;8(8):e021257.

32. Sallis JF, Conway TL, Cain KL, et al. Neighborhood built environment and socioeconomic status in relation to physical activity, sedentary behavior, and weight status of adolescents. *Prev Med (Baltim)*. 2018;110:47-54.

33. Hankey S, Marshall JD. Urban Form, Air Pollution, and Health. *Curr Environ Heal Reports*. 2017;4(4):491-503.

34. Mancus GC, Campbell J. Integrative Review of the Intersection of Green Space and Neighborhood Violence. *J Nurs Scholarsh*. 2018;50(2):117-125.

35. van den Bosch M, Ode Sang A. Urban natural environments as nature-based solutions for improved public health - A systematic review of reviews. *Environ Res*. 2017;158:373-384.

36. Mohit B, Rosen Z, Muennig PA. The impact of urban speed reduction programmes on health system cost and utilities. *Inj Prev*. 2018;24(4):262-266.

37. Izenberg JM, Mujahid MS, Yen IH. Gentrification and binge drinking in California neighborhoods: It matters how long you’ve lived there. *Drug Alcohol Depend*. 2018;188:1-9.

38. Ortiz J, Casquero-Modrego N, Salom J. Health and related economic effects of residential energy retrofitting in Spain. *Energy Policy*. 2019;130:375..

39. Brown V, Moodie M, Carter R. Evidence for associations between traffic calming and safety and active transport or obesity: A scoping review. *J Transp Heal*. 2017;7:23-37.

40. Ambrose A, et al. Better housing, better health in London Lambeth: the Lambeth Housing Standard health impact assessment and cost benefit analysis. 2018:52. http://www4.shu.ac.uk/research/cresr/sites/shu.ac.uk/files/better-housing-better-health-london-lambeth.pdf.

41. Scott SB, Munoz E, Mogle JA, et al. Perceived neighborhood characteristics predict severity and emotional response to daily stressors. *Soc Sci Med*. 2018;200:262-270.

42. Akhter N, Bambra C, Mattheys K, Warren J, Kasim A. Inequalities in mental health and well-being in a time of austerity: Follow-up findings from the Stockton-on-Tees cohort study. *SSM - Popul Heal*. 2018;6:75-84.

43. Fenelon A, Mayne P, Simon AE, et al. Housing Assistance Programs and Adult Health in the United States. *Am J Public Health*. 2017;107(4):571-578..

44. Schnake-Mahl A, Sommers BD, Subramanian S V, Waters MC, Arcaya M. Effects of gentrification on health status after Hurricane Katrina. *Heal Place*. 2019.

45. Fornell B, Correa M, Lopez Del Amo MP, Martin JJ. Influence of changes in the Spanish labor market during the economic crisis (2007-2011) on perceived health. *Qual Life Res*. 2018;27(8):2095-2105.

46. Zapata-Diomedi B, Veerman JL. The association between built environment features and physical activity in the Australian context: a synthesis of the literature. *BMC Public Health*. 2016;16:484.

47. Quam VGM, Rocklöv J, Quam MBM, Lucas RAI. Assessing greenhouse gas emissions and health co-benefits: A structured review of lifestyle-related climate change mitigation strategies. *Int J Environ Res Public Health*. 2017;14(5).

48. Naik Y, Jones S, Christmas H, et al. Collaborative health impact assessment and policy development to improve air quality in West Yorkshire-A case study and critical reflection. *Climate*. 2017;5(3).

49. Dragan KL, Ellen IG, Glied SA. Gentrification and the health of low-income children in New York city. *Health Aff*. 2019;38(9):1425-1432.

50. Mac McCullough J, Leider JP. The Importance of Health and Social Services Spending to Health Outcomes in Texas, 2010-2016. *South Med J*. 2019;112(2):91-97.

51. Aidala AA, Wilson MG, Shubert V, et al. Housing Status, Medical Care, and Health Outcomes Among People Living With HIV/AIDS: A Systematic Review. *Am J Public Health*. 2016;106(1):e1-e23.

52. Gibney S, Zhang M, Brennan C. Age-friendly environments and psychosocial wellbeing: a study of older urban residents in Ireland. *Aging Ment Heal*. 2019.

53. Bornioli A, Parkhurst G, Morgan PL. Psychological Wellbeing Benefits of Simulated Exposure to Five Urban Settings: an Experimental Study From the Pedestrian’s Perspective. *J Transp Heal*. 2018;9:105-116.

54. McCormack GR, Cabaj J, Orpana H, et al. A scoping review on the relations between urban form and health: a focus on Canadian quantitative evidence. *Heal Promot Chronic Dis Prev Canada*. 2019;39(5):187-200.

55. Rojas-Rueda D, Nieuwenhuijsen MJ, Gascon M, Perez-Leon D, Mudu P. Green spaces and mortality: a systematic review and meta-analysis of cohort studies. *Lancet Planet Heal*. 2019;3(11):e469-e477.

56. Halliday E, Barr B, Higgerson J, Holt V, Ortiz-Nunez A, Ward F. Using local authority entrance charges to tackle inequalities in physical activity? A qualitative study of leisure and public health perspectives. *J Public Health (Bangkok)*. 2018;40(3):567-572.

57. Martenies SE, Milando CW, Batterman SA. Air pollutant strategies to reduce adverse health impacts and health inequalities: a quantitative assessment for Detroit, Michigan. *Air Qual Atmos Heal*. 2018;11(4):409-422.

58. Hernandez D, Phillips D, Siegel EL. Exploring the Housing and Household Energy Pathways to Stress: A Mixed Methods Study. *Int J Environ Res Public Heal Electronic Resour*. 2016;13(9):14.

59. Pega F, Wilson N. A Systematic Review of Health Economic Analyses of Housing Improvement Interventions and Insecticide-Treated Bednets in the Home. *PLoS ONE Electronic Resour*. 2016;11(6):e0151812.

60. Anderson LM, Adeney KL, Shinn C, Safranek S, Buckner‐Brown J, Krause LK. Community coalition‐driven interventions to reduce health disparities among racial and ethnic minority populations. *Cochrane Database Syst Rev*. 2015;(6). http://dx.doi.org/10.1002/14651858.CD009905.pub2.

61. Qureshi ME, Dixon J, Wood M. Public policies for improving food and nutrition security at different scales. *Food Secur*. 2015;7(2):393-403.

62. Ziersch A, Due C. A mixed methods systematic review of studies examining the relationship between housing and health for people from refugee and asylum seeking backgrounds. *Soc Sci Med*. 2018;213:199-219.

63. Alderton A, Villanueva K, O’Connor M, Boulange C, Badland H. Reducing inequities in early childhood mental health: How might the neighborhood built environment help close the gap? a systematic search and critical review. *Int J Environ Res Public Health*. 2019;16(9). https://www.mdpi.com/1660-4601/16/9/1516/pdf.

64. Thornton RL, Glover CM, Cene CW, Glik DC, Henderson JA, Williams DR. Evaluating Strategies For Reducing Health Disparities By Addressing The Social Determinants Of Health. *Health Aff*. 2016;35(8):1416-1423.

65. Peña-García A, Hurtado A, Aguilar-Luzón MC. Impact of public lighting on pedestrians’ perception of safety and well-being. *Saf Sci*. 2015;78:142-148.

66. Sallis JF, Spoon C, Cavill N, et al. Co-benefits of designing communities for active living: an exploration of literature. *Int J Behav Nutr Phys Act*. 2015;12:30.

67. Bradley EH, Canavan M, Rogan E, et al. Variation in health outcomes: The role of spending on social services, public health, and health care, 2000-09. *Health Aff*. 2016;35(5):760-768. <http://content.healthaffairs.org/content/35/5/760.full.pdf>.

***Not place-centred/contextual studies (n=53)***

1. Mohan G, Longo A, Kee F. Evaluation of the health impact of an urban regeneration policy: Neighbourhood Renewal in Northern Ireland. *J Epidemiol Community Health*. 2017;71(9):919-927.

2. Norwood P, Eberth B, Farrar S, Anable J, Ludbrook A. Active travel intervention and physical activity behaviour: an evaluation. *Soc Sci Med*. 2014;113:50-58.

3. Unbehaun W, Gaupp-Berghausen M, Jens P. Walking in vienna: Smoothing the way for creating a new urban lifestyle. *Transp Sustain*. 2017;9:317-346.

4. Goodman A, Panter J, Sharp SJ, Ogilvie D. Effectiveness and equity impacts of town-wide cycling initiatives in England: a longitudinal, controlled natural experimental study. *Soc Sci Med*. 2013;97:228-237.

5. Veerman JL, Zapata-Diomedi B, Gunn L, et al. Cost-effectiveness of investing in sidewalks as a means of increasing physical activity: a RESIDE modelling study. *BMJ Open*. 2016;6(9):e011617.

6. Smelson DA, Perez CK, Farquhar I, Byrne T, Colegrove A. Permanent Supportive Housing and Specialized Co-Occurring Disorders Wraparound Services for Homeless Individuals. *J Dual Diagn*. 2018;14(4):247-256.

7. de Heer HD, Balcazar HG, Wise S, Redelfs AH, Lee Rosentha E, Duarte MO. Improved cardiovascular risk among Hispanic border participants of the M Corazón Mi Comunidad Promotores de Salud Model: The HEART II cohort intervention study 2009-2013. *Front Public Heal*. 2015;3.

8. Voßemer J, Gebel M, Kadri T, Unt M, Högberg B, Strandh M. The Effects of Unemployment and Insecure Jobs on Well-Being and Health: The Moderating Role of Labor Market Policies. *Soc Indic Res*. 2018;138(3):1229-1257.

9. Heinen E, Panter J, Mackett R, Ogilvie D. Changes in mode of travel to work: A natural experimental study of new transport infrastructure. *Int J Behav Nutr Phys Act*. 2015;12(1).

10. Goodman A, Sahlqvist S, Ogilvie D, iConnect C. New walking and cycling routes and increased physical activity: one- and 2-year findings from the UK iConnect Study. *Am J Public Health*. 2014;104(9):e38-46.

11. Sahlqvist S, Goodman A, Jones T, et al. Mechanisms underpinning use of new walking and cycling infrastructure in different contexts: mixed-method analysis. *Int J Behav Nutr Phys Act*. 2015;12:24.

12. Powell C, Ellasante I, Korchmaros JD, Haverly K, Stevens S. iTEAM: Outcomes of an Affirming System of Care Serving LGBTQ Youth Experiencing Homelessness. *Fam Soc*. 2016;97(3):181.

13. Song A V, Dutra LM, Neilands TB, Glantz SA. Association of Smoke-Free Laws With Lower Percentages of New and Current Smokers Among Adolescents and Young Adults: An 11-Year Longitudinal Study. *JAMA Pediatr*. 2015;169(9):e152285.

14. Hillier-Brown F, Thomson K, Mcgowan V, et al. The effects of social protection policies on health inequalities: Evidence from systematic reviews. *Scand J Public Health*. 2019;47(6). doi:10.1177/1403494819848276

15. Rose D. The impact of active labour market policies on the well-being of the unemployed. *J Eur Soc Policy*. 2019;29(3):396-410..

16. McNamara B, Rosenwax L, Lee EA, Same A. Evaluation of a healthy ageing intervention for frail older people living in the community. *Australas J Ageing*. 2016;35(1):30-35.

17. Bagnall AM, Radley D, Jones R, et al. Whole systems approaches to obesity and other complex public health challenges: a systematic review. *BMC Public Health*. 2019;19(1):8.

18. Puig-Barrachina V, Giro P, Artazcoz L, et al. The impact of Active Labour Market Policies on health outcomes: a Scoping review. *Eur J public Heal*. 2019;25.

19. Afshin A, Penalvo J, Del Gobbo L, et al. CVD Prevention Through Policy: a Review of Mass Media, Food/Menu Labeling, Taxation/Subsidies, Built Environment, School Procurement, Worksite Wellness, and Marketing Standards to Improve Diet. *Curr Cardiol Rep*. 2015;17(11):98.

20. Baum F, Delany-Crowe T, MacDougall C, et al. To what extent can the activities of the South Australian Health in All Policies initiative be linked to population health outcomes using a program theory-based evaluation? *BMC Public Health*. 2019;19(1):88.

21. McDaid D, Park AL. Investing in mental health and well-being: findings from the DataPrev project. *Health Promot Int*. 2011;26:i108-39..

22. Mehdipanah R, Marra G, Melis G, Gelormino E. Urban renewal, gentrification and health equity: a realist perspective. *Eur J Public Health*. 2018;28(2):243-248.

23. Tan CE, Glantz SA. Association between smoke-free legislation and hospitalizations for cardiac, cerebrovascular, and respiratory diseases: a meta-analysis. *Circulation*. 2012;126(18):2177-2183.

24. Lal A, Moodie M, Abbott G, et al. The impact of a park refurbishment in a low socioeconomic area on physical activity: a cost-effectiveness study. *Int J Behav Nutr Phys Act*. 2019;16(1):26.

25. Britton E, Kindermann G, Domegan C, Carlin C. Blue care: a systematic review of blue space interventions for health and wellbeing. *Health Promot Int*. 2020;35(1):50-69.

26. Slopen N, Fenelon A, Newman S, Boudreaux M. Housing Assistance and Child Health: A Systematic Review. *Pediatrics*. 2018;141(6):1-14.

27. Reinhard E, Carrino L, Courtin E, van Lenthe FJ, Avendano M. Public Transportation Use and Cognitive Function in Older Age: A Quasiexperimental Evaluation of the Free Bus Pass Policy in the United Kingdom. *Am J Epidemiol*. 2019;188(10):1774-1783.

28. Brown V, Moodie M, Cobiac L, Mantilla H, Carter R. Obesity-related health impacts of active transport policies in Australia - a policy review and health impact modelling study. *Aust New Zeal J Public Heal*. 2017;41(6):611-616..

29. Patterson R, Webb E, Mindell JS, Millett C, Laverty AA. Ethnic group differences in impacts of free bus passes in England: A national study. *J Transp Heal*. 2018;11:1-14.

30. Whitley E, Craig P, Popham F. Impact of the statutory concessionary travel scheme on bus travel among older people: A natural experiment from England. *Ageing Soc*. 2019.

31. Robinson T, Brown H, Norman PD, Fraser LK, Barr B, Bambra C. The impact of New Labour’s English health inequalities strategy on geographical inequalities in infant mortality: a time-trend analysis. *J Epidemiol Community Health*. 2019;73(6):564-568.

32. Winters M, Buehler R, Gotschi T. Policies to Promote Active Travel: Evidence from Reviews of the Literature. *Curr Environ Heal Reports*. 2017;4(3):278-285.

33. Kessler RC, Duncan GJ, Gennetian LA, et al. Associations of housing mobility interventions for children in high-poverty neighborhoods with subsequent mental disorders during adolescence. *JAMA*. 2014;311(9):937-948.

34. Ogilvie D, Griffin S, Jones A, et al. Commuting and health in Cambridge: a study of a “natural experiment” in the provision of new transport infrastructure. *BMC Public Health*. 2010;10:703.

35. Pfeiffer D. Rental Housing Assistance and Health: Evidence From the Survey of Income and Program Participation. *Hous Policy Debate*. 2018;28(4):515-533.

36. Ettinger de Cuba S, Chilton M, Bovell-Ammon A, et al. Loss Of SNAP Is Associated With Food Insecurity And Poor Health In Working Families With Young Children. *Health Aff*. 2019;38(5):765-773.

37. Molenberg FJM, Panter J, Burdorf A, van Lenthe FJ. A systematic review of the effect of infrastructural interventions to promote cycling: strengthening causal inference from observational data. *Int J Behav Nutr Phys Act*. 2019;16(1):93..

38. Husk K, Lovell R, Cooper C, Stahl-Timmins W, Garside R. Participation in environmental enhancement and conservation activities for health and well-being in adults: a review of quantitative and qualitative evidence. *Cochrane Database Syst Rev*. 2016;(5):CD010351.

39. Malberg Dyg P, Christensen S, Peterson CJ. Community gardens and wellbeing amongst vulnerable populations: a thematic review. *Heal Promot Int*. 2019;1.

40. Smelson DA, Zaykowski H, Guevermont N, et al. Integrating Permanent Supportive Housing and Co-Occurring Disorders Treatment for Individuals Who Are Homeless. *J Dual Diagn*. 2016;12(2):193-201.

41. Salvo D, Banda JA, Sheats JL, Winter SJ, Lopes Dos Santos D, King AC. Impacts of a Temporary Urban Pop-Up Park on Physical Activity and Other Individual- and Community-Level Outcomes. *J Urban Heal*. 2017;94(4):470-481.

42. Carnemolla P, Bridge C. Accessible housing and health-related quality of life: Measurements of wellbeing outcomes following home modifications. *Archnet-IJAR*. 2016;10(2):38-51.

43. Pollack CE, Du S, Blackford AL, Thornton R, DeLuca S, Herring B. What are the effects of neighborhood poverty on healthcare utilization? Evidence from the moving to opportunity experiment. *J Gen Intern Med*. 2018;33:397.

44. Mason TG, Schooling CM, Chan KP, Tian L. An evaluation of the air quality health index program on respiratory diseases in Hong Kong: An interrupted time series analysis. Atmos Environ. 2019;211:151-158.

45. Franse CB, van Grieken A, Alhambra-Borrás T, et al. The effectiveness of a coordinated preventive care approach for healthy ageing (UHCE) among older persons in five European cities: A pre-post controlled trial. *Int J Nurs Stud*. 2018;88:153-162.

46. Guzman A, Walsh MC, Smith SS, Malecki KC, Nieto FJ. Evaluating effects of statewide smoking regulations on smoking behaviors among participants in the Survey of the Health of Wisconsin. *WMJ*. 2012;111(4):166-171; quiz 172.

47. Martin A, Suhrcke M, Ogilvie D. Financial incentives to promote active travel: an evidence review and economic framework. *Am J Prev Med*. 2012;43(6):e45-57.

48. Egerer MH, Philpott SM, Bichier P, Jha S, Liere H, Lin BB. Gardener well-being along social and biophysical landscape gradients. *Sustain*. 2018;10(1)..

49. Hirsch JA, Meyer KA, Peterson M, Zhang L, Rodriguez DA, Gordon-Larsen P. Municipal investment in off-road trails and changes in bicycle commuting in Minneapolis, Minnesota over 10 years: a longitudinal repeated cross-sectional study. *Int J Behav Nutr Phys Act*. 2017;14(1):21.

50. McAllister A, Almroth M, Harber-Aschan L, Larsson S, Burström B, Fritzell S. How do macro-level structural determinants affect inequalities in mental health? – a systematic review of the literature. *Int J Equity Health*. 2018;17(1).

51. Kramer D, Droomers M, Jongeneel-Grimen B, Wingen M, Stronks K, Kunst AE. The impact of area-based initiatives on physical activity trends in deprived areas; a quasi-experimental evaluation of the Dutch District Approach. *Int J Behav Nutr Phys Act*. 2014;11(1):36.

52. RA S-F, Strogatz D, ML G, et al. The Strong Hearts, Healthy Communities Program 2.0: An RCT Examining Effects on Simple 7. *Am J Prev Med*. 2020;59(1):32-40.

53. Pullyblank K, Strogatz D, SC F, et al. Effects of the Strong Hearts, Healthy Communities Intervention on Functional Fitness of Rural Women. *J Rural Heal Off J Am Rural Heal Assoc Natl Rural Heal Care Assoc*. 2020;36(1):104-110.

***No health/health inequalities/social outcomes (n=18)***

1. Reddy AL, Gomez M, Dixon SL. The New York State Healthy Neighborhoods Program: Findings From an Evaluation of a Large-Scale, Multisite, State-Funded Healthy Homes Program. *J Public Heal Manag Pract*. 2017;23(2):210-218.

2. Vardoulakis S, Kettle R, Cosford P, et al. Local action on outdoor air pollution to improve public health. *Int J Public Health*. 2018;63(5):557-565.

3. Weber AM, Trojan J. The Restorative Value of the Urban Environment: A Systematic Review of the Existing Literature. *Environ Health Insights*. 2018;12:1.

4. Zellmer L, Fleming N. Wheels For All: Addressing Social Determinants of Health One Bicycle at a Time. *WMJ*. 2017;116(3):165-167.

5. Droomers M, Harting J, Jongeneel-Grimen B, Rutten L, van Kats J, Stronks K. Area-based interventions to ameliorate deprived Dutch neighborhoods in practice: does the Dutch District Approach address the social determinants of health to such an extent that future health impacts may be expected? *Prev Med (Baltim)*. 2014;61:122-127.

6. Thurber A, Bohmann Claire R, Heflinger CA. Spatially integrated and socially segregated: The effects of mixed-income neighbourhoods on social well-being. *Urban Stud*. 2018;55(9):1859-1874.

7. Smith M, Hosking J, Woodward A, et al. Systematic literature review of built environment effects on physical activity and active transport - an update and new findings on health equity. *Int J Behav Nutr Phys Act*. 2017;14(1):158.

8. Cheadle A, Samuels SE, Rauzon S, et al. Approaches to measuring the extent and impact of environmental change in three California community-level obesity prevention initiatives. *Am J Public Health*. 2010;100(11):2129-2136.

9. Wang L, Zhong B, Vardoulakis S, et al. Air quality strategies on public health and health equity in Europe—A systematic review. *Int J Environ Res Public Health*. 2016. doi:10.3390/ijerph13121196

10. Houghton A, Castillo-Salgado C. Health co-benefits of green building design strategies and community resilience to urban flooding: A systematic review of the evidence. *Int J Environ Res Public Health*. 2017;14(12). http://www.mdpi.com/1660-4601/14/12/1519/pdf.

11. McFadden A, Siebelt L, Gavine A, et al. Gypsy, Roma and Traveller access to and engagement with health services: a systematic review. *Eur J Public Health*. 2018;28(1):74-81.

12. Philbin MM, Parker CM, Flaherty MG, Hirsch JS. Public Libraries: A Community-Level Resource to Advance Population Health. *J Community Health*. 2019;44(1):192-199.

13. Oates GR, Hamby BW, Bae S, Norena MC, Hart HO, Fouad MN. Bikeshare use in urban communities: Individual and neighborhood factors. Ethn Dis. 2017;27:303-312. https://www.ethndis.org/edonline/index.php/ethndis/article/view/787/935.

14. Chisholm E, Pierse N, Davies C, Howden‐Chapman P, Howden-Chapman P. Promoting health through housing improvements, education and advocacy: Lessons from staff involved in Wellington’s Healthy Housing Initiative. *Heal Promot J Aust*. 2020;31(1):7-15.

15. Snyder J, Kanekar A, Prince B. Growing Healthy Communities Initiative: Transforming the Built Environment to Combat Obesity. *Californian J Health Promot*. 2018;16(2):57-65.

16. Jacobs DE, Tobin M, Targos L, et al. Replacing Windows Reduces Childhood Lead Exposure: Results From a State-Funded Program. *J Public Heal Manag Pract*. 2016;22(5):482-491.

17. Cheadle A, Cromp D, Krieger JW, et al. Promoting Policy, Systems, and Environment Change to Prevent Chronic Disease: Lessons Learned From the King County Communities Putting Prevention to Work Initiative. *J Public Heal Manag Pract*. 2016;22(4):348-359.

18. JLC L, TLT L, RTH H. Understanding Outdoor Gyms in Public Open Spaces: A Systematic Review and Integrative Synthesis of Qualitative and Quantitative Evidence. *Int J Environ Res Public Health*. 2018;15(4). https://pubmed.ncbi.nlm.nih.gov/29587402/.

***Conference proceedings/protocols (n=18)***

1. Gusmano MK, Rodwin VG, Weisz D. Medicare Beneficiaries Living In Housing With Supportive Services Experienced Lower Hospital Use Than Others. *Health Aff*. 2018;37(10):1562-1569.

2. Nct. Strong Hearts for New York: a Rural Heart Disease Prevention Study. *https://clinicaltrials.gov/show/NCT03059472*. 2017.

3. Hammink C, Moor N, Mohammadi M. A systematic literature review of persuasive architectural interventions for stimulating health behaviour. *Facilities*. 2019;37(11):743-761.

4. Ward M, Gibney S, O’Callaghan D, Shannon S. Age-friendly environments, active lives? A study of physical activity among older adults in Ireland. *Age Ageing Conf 66th Annu Sci Meet Irish Gerontol Soc Transform Ageing Across Borders Ireland*. 2018;47.

5. Campbell TR, Roberts NJ. The impacts low emission zones have on improving health and decreasing health inequalities. *Thorax*. 2019;74:A171-A172.

6. Houle J, Coulombe S, Radziszewski S, et al. An intervention strategy for improving residential environment and positive mental health among public housing tenants: rationale, design and methods of Flash on my neighborhood! *BMC Public Health*. 2017;17(1):737.

7. Prins RG, Kamphuis CB, de Graaf JM, Oenema A, van Lenthe FJ. Physical and social environmental changes to promote walking among Dutch older adults in deprived neighbourhoods: the NEW.ROADS study. *BMC Public Health*. 2016;16:907.

8. Nct. Impact of BC Farmers’ Market Nutrition Coupon Program on Diet Quality and Psychosocial Well-being of Low-income Adults. *https://clinicaltrials.gov/show/NCT03952338*. 2019.

9. Macmillan AK, Mackie H, Hosking JE, et al. Controlled before-after intervention study of suburb-wide street changes to increase walking and cycling: Te Ara Mua-Future Streets study design. *BMC Public Health*. 2018;18(1):850.

10. Bowyer S. “The shop is more than a shop”: Listening to the ‘Local Voice’ to illustrate the connection of health and place, when considering food access in a rural island community. *Rev Esp Nutr Humana y Diet*. 2016;20:526-527.

11. Chandra PS, Lakshmi S, Nanjundaswamy MH, Shiva L. The impact of urbanization on mental health in India. *Curr Opin Psychiatry*. 2018;31(3):276-281.

12. Hendricks K, Wilkerson R, Vogt C, TenBrink S. Transforming a small midwestern city for physical activity: from the sidewalks up. *J Phys Act Health*. 2009;6(6):690-698.

13. Hurst G, Davey R, Smith G, Kurth J. Results from a community-led intervention aimed at reducing health inequalities in Stoke-on-Trent, UK. *J Sci Med Sport*. 2012;15:S260.

14. Jamaludin M, Nazar GP, Palladino R, Tsakos G, Watt RG, Millett C. Smoke-free legislation and socioeconomic inequalities in smoking-related morbidity and mortality among adults: A systematic review. *Tob Induc Dis*. 2018;16:148.

15. Gittelsohn J, Trude A. Environmental interventions for obesity and chronic disease prevention. *J Nutr Sci Vitaminol (Tokyo)*. 2015;61:S15-S16.

16. Telfar-Barnard L, Bennett J, Howden-Chapman P, et al. Measuring the Effect of Housing Quality Interventions: The Case of the New Zealand “Rental Warrant of Fitness.” *Int J Environ Res Public Heal Electronic Resour*. 2017;14(11):7.

17. Eggleton K, Mc Kay K. Developing a sense of place: Creating a home through a healthy homes initiative. *Aust J Prim Health*. 2019;25:xiii..

18. Hobin E, Swanson A, Booth G, et al. Physical activity trails in an urban setting and cardiovascular disease morbidity and mortality in Winnipeg, Manitoba, Canada: a study protocol for a natural experiment. *BMJ Open*. 2020;10(2):e036602.

***DARE Criteria not met (n=3)***

1. Newman L, Baum F, Javanparast S, O’Rourke K, Carlon L. Addressing social determinants of health inequities through settings: a rapid review. *Health Promot Int*. 2015;30:ii126-43.

2. Benmarhnia T, Rey L, Cartier Y, Clary CM, Deguen S, Brousselle A. Addressing equity in interventions to reduce air pollution in urban areas: a systematic review. *Int J Public Health*. 2014;59(6):933-944.

3. Hollederer A. Health promotion and prevention among the unemployed: a systematic review. *Health Promot Int*. 2019;34(6):1078-1096.

***Not high income country (n=2)***

1. Hirsch JA, DeVries DN, Brauer M, Frank LD, Winters M. Impact of new rapid transport on physical activity: a meta-analysis. *Preventive Medicine Reports*. 2018;10: 184-190.

2. Foster C, Kelly P, Reid HAB, Roberts N, Murtagh EM, Humpreys DK, Panter J, Milton K. What works to promote walking at the population level? A systematic review. *Br J Sports Med.* 2018;52:807-812.

1. If there fewer than five studies that are relevant, these can be included in full here. However if there are more than five, studies should be grouped if interventions are similar. [↑](#footnote-ref-1)
2. Setting also includes place-based factor – social, physical or economic environment – in addition to country/region [↑](#footnote-ref-2)
3. As measured by systematic review authors. [↑](#footnote-ref-3)
